# Progressive multifocal leukoencephalopathy genetic risk variants for pharmacovigilance of immunosuppressant therapies

**DOI:** 10.3389/fneur.2022.1016377

**Published:** 2022-12-14

**Authors:** Eli Hatchwell, Edward B. Smith, Shapour Jalilzadeh, Christopher D. Bruno, Yassine Taoufik, Houria Hendel-Chavez, Roland Liblau, David Brassat, Guillaume Martin-Blondel, Heinz Wiendl, Nicholas Schwab, Irene Cortese, Maria Chiara Monaco, Luisa Imberti, Ruggero Capra, Jorge R. Oksenberg, Jacques Gasnault, Bruno Stankoff, Todd A. Richmond, David M. Rancour, Igor J. Koralnik, Barbara A. Hanson, Eugene O. Major, Christina R. Chow, Peggy S. Eis

**Affiliations:** ^1^Population Bio UK, Inc., Oxfordshire, United Kingdom; ^2^Population Bio, Inc., New York, NY, United States; ^3^Emerald Lake Safety LLC, Newport Beach, CA, United States; ^4^Department of Hematology and Immunology, Hôpitaux Universitaires Paris-Saclay and INSERM 1186, Institut Gustave Roussy, Villejuif, France; ^5^Infinity, Université Toulouse, CNRS, INSERM, UPS, Toulouse, France; ^6^Department of Immunology, CHU Toulouse, Hôpital Purpan, Toulouse, France; ^7^Department of Infectious and Tropical Diseases, Toulouse University Hospital Center, Toulouse, France; ^8^Department of Neurology With Institute of Translational Neurology, University Hospital Münster, Münster, Germany; ^9^Experimental Immunotherapeutics Unit, National Institute of Neurological Disorders and Stroke, National Institutes of Health, Bethesda, MD, United States; ^10^Viral Immunology Section, National Institute of Neurological Disorders and Stroke, National Institutes of Health, Bethesda, MD, United States; ^11^Centro di Ricerca Emato-Oncologica AIL (CREA) and Diagnostic Department, ASST Spedali Civili of Brescia, Brescia, Italy; ^12^Lombardia Multiple Sclerosis Network, Brescia, Italy; ^13^Department of Neurology, Weill Institute for Neurosciences, University of California, San Francisco, San Francisco, CA, United States; ^14^Department of Internal Medicine, Hôpitaux Universitaires Paris-Sud, Le Kremlin-Bicêtre, France; ^15^Department of Neurology, Hôpital Saint-Antoine, Paris, France; ^16^Richmond Bioinformatics Consulting, Seattle, WA, United States; ^17^Lytic Solutions, LLC, Madison, WI, United States; ^18^Department of Neurology, Feinberg School of Medicine, Northwestern University, Chicago, IL, United States; ^19^Laboratory of Molecular Medicine and Neuroscience, National Institute of Neurological Disorders and Stroke, National Institutes of Health, Bethesda, MD, United States

**Keywords:** immunodeficiency, JC virus, multiple sclerosis, natalizumab, pharmacovigilance, progressive multifocal leukoencephalopathy, PML, serious adverse event

## Abstract

**Background:**

Progressive multifocal leukoencephalopathy (PML) is a rare and often lethal brain disorder caused by the common, typically benign polyomavirus 2, also known as JC virus (JCV). In a small percentage of immunosuppressed individuals, JCV is reactivated and infects the brain, causing devastating neurological defects. A wide range of immunosuppressed groups can develop PML, such as patients with: HIV/AIDS, hematological malignancies (e.g., leukemias, lymphomas, and multiple myeloma), autoimmune disorders (e.g., psoriasis, rheumatoid arthritis, and systemic lupus erythematosus), and organ transplants. In some patients, iatrogenic (i.e., drug-induced) PML occurs as a serious adverse event from exposure to immunosuppressant therapies used to treat their disease (e.g., hematological malignancies and multiple sclerosis). While JCV infection and immunosuppression are necessary, they are not sufficient to cause PML.

**Methods:**

We hypothesized that patients may also have a genetic susceptibility from the presence of rare deleterious genetic variants in immune-relevant genes (e.g., those that cause inborn errors of immunity). In our prior genetic study of 184 PML cases, we discovered 19 candidate PML risk variants. In the current study of another 152 cases, we validated 4 of 19 variants in both population controls (gnomAD 3.1) and matched controls (JCV+ multiple sclerosis patients on a PML-linked drug ≥ 2 years).

**Results:**

The four variants, found in immune system genes with strong biological links, are: *C8B*, 1-57409459-C-A, rs139498867; *LY9* (alias *SLAMF3*), 1-160769595-AG-A, rs763811636; *FCN2*, 9-137779251-G-A, rs76267164; *STXBP2*, 19-7712287-G-C, rs35490401. Carriers of any one of these variants are shown to be at high risk of PML when drug-exposed PML cases are compared to drug-exposed matched controls: P value = 3.50E-06, OR = 8.7 [3.7–20.6]. Measures of clinical validity and utility compare favorably to other genetic risk tests, such as *BRCA1* and *BRCA2* screening for breast cancer risk and HLA-B^*^15:02 pharmacogenetic screening for pharmacovigilance of carbamazepine to prevent Stevens-Johnson Syndrome and Toxic Epidermal Necrolysis.

**Conclusion:**

For the first time, a PML genetic risk test can be implemented for screening patients taking or considering treatment with a PML-linked drug in order to decrease the incidence of PML and enable safer use of highly effective therapies used to treat their underlying disease.

## Introduction

Progressive multifocal leukoencephalopathy (PML) is a rare brain disease caused by the reactivation of JC virus (JCV) in immunosuppressed individuals. As an aggressive demyelinating disorder, PML can be fatal and is often severe and debilitating; almost 70% of survivors experience ongoing neurological disability and there is no approved treatment once PML develops (1). While PML is quite rare, infection with JCV is common, with most patients being asymptomatic. Based on serological testing, JCV has an estimated worldwide prevalence of 40–70% (2). More recent studies in Asian populations showed even higher rates of seropositivity, ranging from 70 to 80% (3–5). We note that JCV is formally named human polyomavirus 2 (HPyV-2 or HuPyV2) (6, 7) but, for simplicity, will be referred to as JCV in the present study.

Immunosuppression in JCV-seropositive (JCV+) individuals that develop PML can be due to a wide range of underlying diseases and/or drugs but is broadly related to three underlying disease states (1, 8): Human Immunodeficiency Virus (HIV)-infected, hematological malignancies (i.e., lymphoproliferative diseases such as leukemias, lymphomas, and multiple myeloma); and autoimmune disorders, such as rheumatoid arthritis (RA) and systemic lupus erythematosus (SLE). HIV-infected acquired immunodeficiency syndrome (AIDS) patients represent the largest proportion of PML cases (~50%). Rates in this population substantially dropped after the 1996 introduction of highly active antiretroviral therapy (HAART), although at least 10% of HIV patients who are considered “immunological non-responders” to antiviral therapies (9) could continue to have elevated PML risk similar to the pre-1996 era. Conversely, iatrogenic PML (i.e., resulting from drug exposure) is on the rise with the growing number of immunosuppressant therapies used to treat various immune disorders (1, 10). Historically, iatrogenic PML risk was sufficiently high for psoriasis patients taking efalizumab (brand name Raptiva) that the therapy was withdrawn from the market worldwide in 2009 based upon a PML incidence rate of 0.158% or ~16 in 10,000 (11). Today, multiple sclerosis (MS) patients on disease-modifying therapies are the largest proportion of iatrogenic PML cases (1).

There are over three dozen drugs that include a PML warning in their prescribing information (USA) and/or mention PML in their product characteristics (European Medicines Agency). Examples include alemtuzumab, brentuximab vedotin, dimethyl fumarate, efalizumab, fingolimod, ibrutinib, and natalizumab; as well as anti-CD20 antibodies (also known as B cell depletion therapies) such as obinutuzumab, ocrelizumab, ofatumumab, and rituximab (12, 13). Of recent note, the PML warning in ocrelizumab's prescribing information was substantially expanded in August 2022.

Given the large number of drugs linked to the development of PML (1, 10, 14, 15), it is critical to identify additional risk factors that can be taken into consideration when patients and their clinicians are selecting a therapy for treatment of the underlying disorder. Since JCV infection is a requirement for developing PML (although most JCV-infected individuals will not develop PML), testing patients with a JCV antibody test (including assessing their index level) can be useful for informing PML risk (16, 17). For example, testing every 6 months (18) is recommended by the European Medicines Agency for patients on natalizumab who are JCV-negative or have a low index value. However, development of other PML risk biomarkers continues to be an area of high unmet need (19), especially since the specificity of the JCV antibody test is low (40–70% of the population are seropositive for JCV) (2, 20), the test's false negative rate is reported to be 3% (manufacturer's prescribing information for natalizumab, Dec-2021), and index levels may be unreliable for anti-CD20 therapies because of their mechanism of action (i.e., reduced antibody levels may result in lower anti-JCV antibody levels) (21, 22). Another suggested biomarker is serum neurofilament light chain (NfL) levels (23, 24), but it is only useful in verifying PML onset and resolution of the disease (in natalizumab-treated MS patients) as opposed to predicting who may get PML in the future (i.e., before a patient decides to take a PML-linked therapy). This is an important distinction given the seriousness of the condition and its limited treatment options once it develops.

Host genetic predisposition to PML (i.e., an individual has one or more genetic variants in their genome that increases their risk of developing PML) was proposed to be a significant risk factor (25); see also Mills and Mao-Draayer (26). This hypothesis is supported by a growing number of PML case reports (25, 27–42) in which the patients were found to have mutation(s) in known immunodeficiency disorder genes (43, 44). We previously explored the possibility of genetic predisposition to PML in the largest genetic study to date, whole-exome sequencing (WES) of 184 PML cases (45). That work identified 19 rare genetic variants in known immune-modulating genes that were significantly more common in PML patients compared to populations in the Genome Aggregation Database (gnomAD) database (46).

This study reports the frequency of these variants in additional PML cases (152 new, 336 total). By far, this is the largest ever assembled set of DNA samples from PML cases, providing a unique resource for studying germline genetic links to the disease. Importantly, this work, for the first time, compares 110 drug-exposed PML cases to 718 drug-exposed controls who took PML-linked drugs for ≥2 years. In this case-control analysis, four variants show a particularly strong association with PML; two of these variants only appear in cases and are never observed in the drug-exposed controls.

Due to the severity of PML as a serious adverse event, eight currently marketed drugs have PML in a Boxed Warning in their prescribing information (the FDA's strongest drug label warning) and numerous other drugs have a warning about PML in their product labeling in the USA and similar warnings in the EU, while one drug was withdrawn from the market due to its PML risk. Our reported measures of clinical validity and utility for the identified four variants show that utilization of a simple and inexpensive genotyping test in patients considering treatment with PML-linked immunosuppressant therapies has the potential to reduce the incidence of PML and save lives.

## Methods

### IRB approvals

Written informed consent was obtained from all patients (PML cases and MS controls) participating in this study under IRB approved protocols from the following institutions: Accelerated Cure Project, Comitato Etico Provinciale of Brescia (PI Imberti), Beth Israel Deaconess Medical Center (PI Koralnik), Icahn School of Medicine at Mount Sinai (BioMe Biobank), NINDS/NIH (PIs Major and Cortese), Paris-Sud/INSERM (PI Taoufik), University of California San Francisco (PI Oksenberg), University of Münster (PIs Schwab and Wiendl), Université Toulouse (PIs Brassat, Martin-Blondel, and Liblau), and Vanderbilt University (BioVU Biobank).

### PML cases

In addition to the 184 cases previously studied using WES (45), new cases were assembled for genetic validation via genotyping. A total of 156 new DNA samples were collected from the following collaborating institutions: Accelerated Cure Project (*n* = 1), Comitato Etico Provinciale of Brescia (*n* = 11), NINDS/NIH (*n* = 32), Paris-Sud/INSERM (*n* = 9), Université Toulouse (*n* = 57), University of Münster (*n* = 44), and University of California San Francisco (*n* = 2). Potential cases were assessed using the consensus PML diagnostic criteria (47) and only “Definite” or “Probable” PML cases were retained. Wherever possible, drug exposures for immunosuppressant drugs (approved or used off-label) were noted. Primary underlying diseases were recorded and were then categorized as blood cancer (BC), HIV, MS, or Other. The BC subgroup includes: acute myeloid leukemia, anaplastic plasmacytoma, B-cell lymphoma, chronic lymphocytic leukemia, follicular lymphoma, Hodgkin lymphoma, leukemia, lymphoma, marginal zone lymphoma, myelodysplastic syndrome, multiple myeloma, and non-Hodgkin lymphoma. The Other subgroup includes: alcoholic cirrhosis, anti-synthetase syndrome, aplastic anemia, B-cell deficiency, Behcet's disease, cancer (non-hematological: colon and liver), common variable immunodeficiency, dermatomyositis, dermatopolymyositis, granulomatosis, idiopathic CD4 lymphocytopenia, immune thrombocytopenia, lymphopenia, microscopic polyangiitis, ocular pemphigoid, polycythemia vera, primary CD8 lymphopenia, psoriasis, RA, sarcoidosis (kidney, pulmonary, and unspecified), severe combined immunodeficiency, silicosis, thymoma with immunodeficiency, transplants (bone marrow, kidney, and liver), vasculitis, or unknown.

### Control subjects

For comparison to drug-exposed PML cases, a set of drug-exposed controls with MS (called “matched controls”) were assembled from two laboratories: Université Toulouse and University of California San Francisco (UCSF). Inclusion criteria for controls were as follows: 1) JCV seropositivity, 2) exposure to an immunosuppressant/PML-linked drug for at least 2 years as PML risk increases after 2 years in MS patients (17), and 3) absence of a PML diagnosis. The JCV antibody status was already determined to be positive for all Université Toulouse controls; for the UCSF controls, JCV antibody status experiments were performed by Lytic Solutions (Madison, WI, USA). Detection of anti-JCV IgGs in serum samples was performed according to manufacturer instructions using the ELISA-VIDITEST anti-JCV IgG diagnostic kit (Catalog # ODZ-450) from Vidia spol. s.r.o. (Vestec, Czech Republic; distributed by Boca Scientific Inc., Dedham, MA, USA). All serum samples (diluted 1:100) were run in duplicate and 96-well plates included control human serum samples of known JCV infection status. After color development (using kit-supplied stop solution), absorbance values at 450 nm (with a reference reading at 650 nm) were measured using a Molecular Devices Spectra Max Plus plate reader (San Jose, CA, USA). Background-subtracted values were averaged for each sample. The qualitative interpretation procedure for data analysis was performed according to the manufacturer instructions using the internal plate calibrator value and the plate lot correction factor. Samples with absorbances lower than 90% of the cut-off value were considered negative and samples with absorbances higher than 110% of the cut-off value were considered positive. Samples with values between these two cut-offs were considered indeterminable. Only JCV+ samples were retained for further analyses and all had MS as their primary disease.

To assess PML risk across all primary disease subgroups (BC, HIV, MS, and Other) in the context of population-level data, we used the most recent version (3.1) of gnomAD (46). This release consists of Whole Genome Sequencing (WGS) data for ~76,000 genomes corresponding to a variety of ethnicities; results are also reported by ethnic subgroups for European (EUR, ~34,000 non-Finnish European genomes), African (AFR, ~21,000 genomes), and EUR plus AFR (~55,000 genomes). In addition to the functional prediction methods PolyPhen and SIFT, gnomAD 3.1 also reports the results for other prediction measures of deleteriousness, such as CADD scores.

### Genetic analyses

Ancestry and duplicate sample analyses were assessed for all PML cases and matched controls using previously described methods (45) with the exception that WGS (0.1x read depth) of newly acquired PML cases and matched controls was performed by Psomagen (Rockville, MD, USA). Ancestry analysis was performed by Gencove (New York, NY, USA) using 0.1x read depth WGS data based on implementation of a supervised version of the STRUCTURE model (48), which is trained on a panel of 7,345 individuals grouped in 49 populations. Primary ethnicities were assigned as AFR or EUR based on the majority percentage of ancestry.

For duplicate sample analyses, the low coverage WGS VCF files from Psomagen were filtered using bcftools (v1.10) to include only biallelic single nucleotide variants (SNVs) with exactly two alleles and PASS quality. The filtered variants were then annotated and evaluated for relatedness using plink (v1.9) and KING software (v2.2.6). Duplicate samples were excluded from further analysis.

Previously published WES data on the 19 PML risk variants (45) was reanalyzed in the context of new PML cases and JCV+ matched controls. We note that one of the previously published 185 PML cases was a bone marrow transplant patient whose DNA sample was acquired post-transplant; therefore we excluded this patient from the present analyses. For new PML cases and controls, the 19 variants were genotyped by a service provider (LGC Genomics, UK) with custom designed assays that use kompetitive allele specific PCR (KASP) chemistry. Sex was confirmed via genotyping.

To verify that previously published PML risk variants (45) were associated with PML and not with MS, we assessed 12 of 19 variants in a large genome-wide association study (GWAS) conducted by the International Multiple Sclerosis Genetics Consortium. This MS study used an exome chip (Illumina HumanExome Beadchip) containing 137,007 genome-wide common (12%) and rare (88%) variants to identify MS-associated loci in 32,367 MS cases vs. 36,012 healthy controls. The seven variants that were not assessed were either not found on the Illumina exome array or were not reported in the study (49).

### Statistical and pharmacogenetic test analyses

Association statistics, Odds Ratio (OR) values and P values (two-tailed Fisher's Exact Test), were calculated as previously described (45). To avoid infinite ORs for variants that were not present in matched controls, 0.5 was added to all cells of the contingency table (50). The 19 previously identified variants for PML risk were evaluated in drug-exposed PML cases compared to drug-exposed controls and gnomAD population controls. Several PML cases had mixed ancestry (i.e., one or more other ethnicities present at >5%). Therefore, statistical analyses using gnomAD population controls included all ethnicities (i.e., all ~76,000 WGS data sets).

Following individual variant association testing, combinations of the highest-risk variants (as identified in the case-control analyses) were explored for use in a panel test. Pharmacogenetic test parameters (clinical validity and clinical utility) for this panel test were calculated using the method of Tonk et al. (51). Clinical validity measures are sensitivity, specificity, positive predictive value (PPV), and negative predictive value (NPV). Clinical utility measures are population attributable fraction (PAF), number needed to treat (NNT), and number needed to genotype (NNG). As the incidence of drug-induced PML varies by drug type/exposure, for the adverse event frequency we used the best-established long term rate reported for JCV+ MS patients on natalizumab, which is 3% (17).

## Results

### Assembly of PML cases and matched controls to further validate candidate PML risk genetic variants

**Figure 1:**
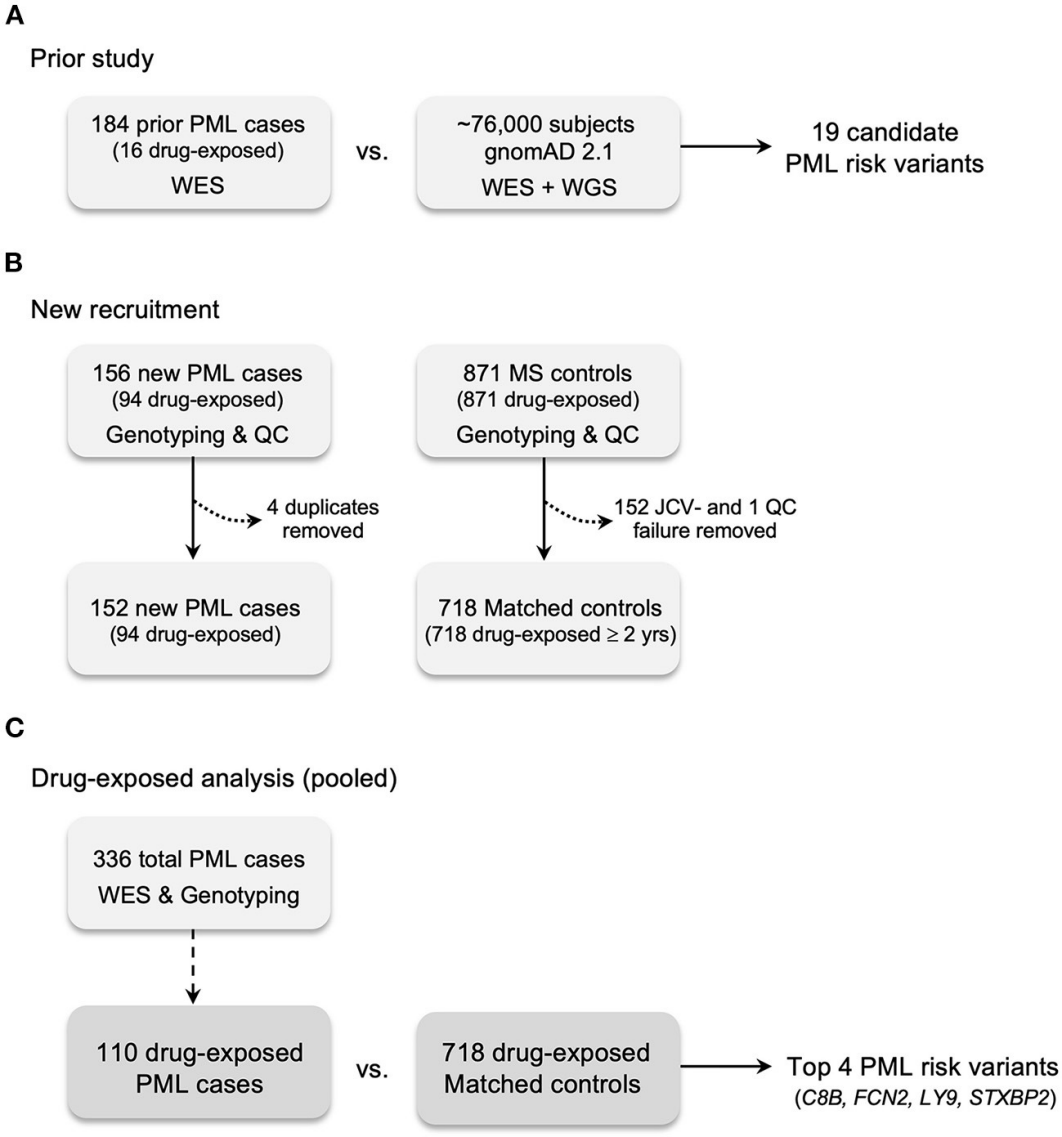
Case and control recruitment and study design. **(A)** Prior study for genetic discovery and validation using Whole Exome Sequencing (WES), 669 candidate immune response genes, and gnomAD 2.1 (WES + WGS) population controls (45). **(B)** New recruitment of PML cases and matched controls (JCV+ MS patients exposed to a PML-linked drug ≥2 years). All PML cases and matched controls were genotyped for the prior study's 19 candidate PML risk variants. Matched controls without JCV serostatus were assayed (see Methods). Excluded cases: four were found to be duplicates of the prior study (see Methods). Excluded controls: 152 JCV seronegative (JCV-) patients; one QC failure for genotyping assays due to low quality DNA. **(C)** Drug-exposed analysis is the pooled subgroup (*n* = 110) of total PML cases (*n* = 336) compared to matched controls. Drug-exposed study results are reported in Tables 2, 5 and genes for the top 4 variants are listed.

A total of 340 potential PML cases were assembled for the present study. Following ancestry and duplicate sample analyses, four newly acquired samples were found to be identical to previous samples and were therefore removed. The final PML cohort includes a total of 336 PML cases: 184 from our previous study (45) and 152 new, unique cases. Using consensus PML diagnostic criteria (47), 287 (85%) cases were Definite PML and 49 (15%) cases were Probable PML. Eleven PML cases (3.3%) had neither predominantly AFR nor EUR ancestry and were assigned EUR. Additionally, 60% (33/55) of AFR and 22% (62/281) of EUR cases had one or more other ethnicities present at >5%. Sex, primary ethnicity, primary disease, and drug exposures for these cases are summarized in Table 1 and a workflow of the recruitment and study design is shown in Figure 1. Of the assembled PML cohort, 110 of 336 (32.7%) PML cases were drug-exposed.

**Table 1:** Summary of PML cases and drug-exposed controls: primary disease group, MS drug exposure, and demographics.

		**Drug-exposed**	
	**Total PML cases**	**PML cases**	**Matched controls^a^**
**Subjects**	336	110	718
**Primary disease^b^**		
HIV	156	0	n/a
MS	94	94	718
Other	45	8	n/a
BC	41	8	n/a
**Drug exposure^c^**		
Natalizumab		86	604
Rituximab		13	25
Unknown^d^		4	0
Dimethyl fumarate		3	43
Alemtuzumab		1	0
Fingolimod		1	55
Glatiramer acetate		1	0
Mycophenolate mofetil		1	0
Ocrelizumab		0	12
TOTAL, non-redundant		110	718
**Sex**		
Male	184	31	194
Female	152	79	524
**Primary ethnicity^e^**		
EUR	281	109	645
AFR	55	1	73

a Drug-exposed matched controls are JCV+ MS patients on an MS drug ≥ 2 years who did not develop PML.

b Primary disease: BC, blood cancer; HIV, human immunodeficiency virus infected; MS, multiple sclerosis; Other, various. See Methods for list of diseases under the BC and Other subgroups.

c All drugs have PML listed in the prescribing information (Boxed Warning and/or Warnings and Precautions) with the exception of glatiramer acetate. Drug exposure times were unavailable for PML cases but are ≥ 2 years for controls (a subset were exposed to two or more drugs for ≥ 2 years). Of the 110 drug-exposed PML cases, four had multiple reported drug exposures: 1 glatiramer acetate (also exposed to interferon beta-1a, but no exposure to natalizumab) and 3 rituximab (also exposed to bendamustine, cyclophosphamide-fludarabine, or cyclosporine-methotrexate-mycophenolate mofetil-steroids-tacrolimus).

d Four PML cases had unknown drug exposures but were assumed to be drug-exposed since all were MS patients, a patient group that is not known to develop PML in the absence of treatment with a disease-modifying therapy.

e A primary ethnicity was assigned as AFR or EUR (see Methods) for statisical analyses. An Other ethnicity was annotated if > 5% of one or more other ethnicities was found: EUR, 62/281 PML cases and 128/645 drug-exposed controls; AFR, 33/55 PML cases and 67/73 drug-exposed controls.

A total of 879 potential controls were assembled from Université Toulouse and UCSF. Of these, 152 samples (all from UCSF) were removed for lack of JCV seropositivity and 9 Toulouse samples were removed for either relatedness or incomplete drug-exposure data. This yielded a final drug-exposed control cohort of 718 individuals, all of whom had MS as a primary disease (hereafter referred to as drug-exposed controls). According to ancestry analysis, 24 (3.3%) controls had neither predominantly AFR nor EUR ancestry and were assigned as EUR (Table 1 and Figure 1).

### Association of top PML risk variants in drug-exposed PML cases vs. matched controls

The presence of the 19 previously identified PML risk variants was assessed in drug-exposed PML cases (*n* = 110) vs. drug-exposed controls (*n* = 718) and vs. gnomAD population controls (*n* = ~76,000). As summarized in Table 2, four variants showed strong association with PML risk in this analysis. Variants in genes *C8B, FCN2*, and *STXBP2* were found to be significant (P value <0.05) compared to both drug-exposed controls and gnomAD population controls. The *LY9* variant was only significant when compared to gnomAD controls, likely a consequence of its very low frequency (11 out of 76,504 subjects). Of note, the *STXBP2* and *LY9* variants were absent in drug-exposed controls and had large effect sizes (OR = 33.1 and 19.6, respectively).

**Table 2:** Association statistics^a^ for PML risk variants: drug-exposed PML cases (*n* = 110) vs. drug-exposed controls and gnomAD 3.1 population controls.

			**Drug-exposed controls^b^**		**gnomAD controls^c^**	
			**(*n* = 718)**		**( *n* = 76,071)**	
**Gene symbol**	**dbSNP ID**	**Variant (GRCh37, hg19)**	**OR (95% CI)**	**P value**	**OR (95% CI)**	**P value**
*STXBP2*	rs35490401	19-7712287-G-C	33.1 (1.6 - 694.4)	**0.0175**	6.8 (1.7 - 27.6)	**0.0373**
*LY9*	rs763811636	1-160769595-AG-A	19.6 (0.8 - 484.3)	0.1330	63.4 (8.1 - 495.5)	**0.0172**
*C8B*	rs139498867	1-57409459-C-A	6.7 (1.7 - 27.3)	**0.0135**	4.3 (1.6 - 11.8)	**0.0159**
*FCN2*	rs76267164	9-137779251-G-A	5.7 (1.7 - 18.8)	**0.0090**	7.0 (2.9 - 17.3)	**0.0001**

a The subset of total PML cases that were drug-exposed (110 of 336) were compared to drug-exposed controls and population controls (gnomAD). P values were calculated using Fisher's Exact Test; OR, odds ratio; CI, confidence interval. Variants are ordered by descending OR and bold-highlighted P values denote significance <0.05.

b Drug-exposed (matched) controls are JCV+ MS patients on a MS drug ≥ 2 years who did not develop PML.

c All ethnicities in gnomAD 3.1 population controls were used (see Methods). Sample size (subject number) varies slightly by variant, n = 76,071 is an average of the 4 variants: *STXBP2*, n = 76,099; *LY9*, n = 76,054; *C8B*, n = 76,081; *FCN2*, n = 76,050.

As summarized in Table 1, natalizumab-exposed PML cases (*n* = 86) represent the largest subgroup of PML cases with a PML-linked drug exposure history in our study. Similarly, natalizumab-exposed controls (*n* = 604) were also the largest subgroup for matched controls. Therefore, we also assessed the association of the PML risk variants in the natalizumab subgroup (see Supplementary Table 6) and found comparable results to the full set of of drug-exposed cases and controls (Table 2 and Supplementary Table 5). The association statistics for three of the four top variants were slightly improved for the natalizumab subgroup (lower P values and higher ORs) but were less significant for the *FCN2* variant.

### PML risk variants are rare and predicted to be pathogenic

All four variants are rare in the general population (Table 3), with gnomAD 3.1 allele frequencies <0.5% and thus providing supporting evidence of their pathogenicity (52). Three variants are missense and are predicted to be probably or possibly damaging by PolyPhen and deleterious by SIFT. The *LY9* variant is a frameshift predicted to cause loss of function of the protein (pLOF). A third prediction method, the CADD score (as reported in gnomAD 3.1), is also reported in Table 3. The CADD score range was 22.8–26.0, indicating that all four variants are predicted to be detrimental (CADD score >20 is the highest category of deleteriousness in gnomAD 3.1 annotation).

**Table 3:** PML risk variant functional impact predictions.^a^

					**gnomAD 3.1** ***in silico*** **predictions**		
**Gene symbol**	**dbSNP ID**	**Variant** **(GRCh37, hg19)**	**gnomAD** **3.1 AF^b^**	**Consequence^c^**	**Polyphen**	**SIFT**	**CADD^e^**
*STXBP2*	rs35490401	19-7712287-G-C	0.001367	missense	probably damaging	deleterious	26.0
*LY9*	rs763811636	1-160769595-AG-A	0.000072	frameshift (pLOF)	n/a^d^	n/a^d^	22.8
*C8B*	rs139498867	1-57409459-C-A	0.004331	missense	possibly damaging	deleterious	23.1
*FCN2*	rs76267164	9-137779251-G-A	0.003393	missense	probably damaging	deleterious	24.0

a These PML risk variants are a subset of the 19 previously reported variants (45). Gray-shading denotes severity of functional predictions: no shading = low impact (none for these 4 variants), light gray, moderate impact, dark gray, high impact.

b AF, allele frequency of the variant in gnomAD 3.1 for all ethnicities (Total).

c Missense variants are amino acid substitutions; for the *LY9* variant, pLOF denotes protein loss-of-function.

d Polyphen and SIFT are prediction methods for missense variants and are not applicable (n/a) to other types of variants (e.g., the high impact frameshift for the *LY9* variant).

e CADD scores >20 are the highest category of deleteriousness in gnomAD 3.1 annotation.

### No association of PML risk variants with MS

Since iatrogenic PML cases are on the rise and MS patients are one of the intended patient groups for a PML risk genetic test, we checked if any of our top four PML risk variants were associated with MS. Previously reported MS genome-wide association study (GWAS) data from a large international study (49) were used for this analysis and included 32,367 MS cases vs. 36,012 healthy controls. Table 4 shows the association results for the top four PML risk variants. Three of the four top variants in genes *C8B, FCN2*, and *STXBP2* show no association with MS. All three had an OR of 1.0 and uncorrected genome-wide P values of 0.07–0.71. The fourth top variant (in the *LY9* gene) is very rare in the general population (gnomAD 3.1 allele frequency = 0.000072) and has not been reported in the literature to be associated with disease (including MS).

**Table 4:** PML risk variant association with MS vs. drug-exposed PML cases.

			**Association with MS^a^**		**Association with PML^b^**	
**Gene symbol**	**dbSNP ID**	**Variant (GRCh37, hg19)**	**OR**	**P value**	**OR**	**P value**
*STXBP2*	rs35490401	19-7712287-G-C	1.0	0.6853	33.1	**0.0175**
*LY9*	rs763811636	1-160769595-AG-A	n/a^c^	n/a^c^	19.6	0.1330
*C8B*	rs139498867	1-57409459-C-A	1.0	0.0670	6.7	**0.0135**
*FCN2*	rs76267164	9-137779251-G-A	1.0	0.7088	5.7	**0.0090**

a MS association results (32,367 MS cases vs. 36,012 healthy controls) were previously reported (49), see Methods for details.

b Drug-exposed results (110 PML cases vs. 718 matched controls) are from Table 2 (as a comparator to the MS association results); P values were calculated using Fisher's Exact Test; OR, odds ratio.

c The *LY9* variant was not evaluated (n/a, not applicable) in the MS association study, likely because it is very rare in the general population (gnomAD 3.1 allele frequency = 0.000072) and therefore not included on the exome chip (Illumina Exome BeadChip).

### Utilization of a genetic risk test to reduce the incidence of PML with immunosuppressant therapies

Based on the results of the association analysis in the drug-exposed PML cases, a panel of four rare variants in genes (*C8B, FCN2, STXBP2*, and *LY9*) with strong immune-linked biology was identified as being potentially useful to identify patients at high risk of PML (see Supplementary Table 9 for analysis of the four individual variants vs. the 4-variant panel test in three different groups of PML cases: All, any Drug-exposed, and Natalizumab-exposed). Clinical validity and population impact measures (i.e., clinical utility) are shown in Table 5. No subject in either cases or controls presented with more than one of the four variants in the panel. Presence of any one of these four variants was 10.9% in the drug-exposed PML cases vs. only 1.4% in the drug-exposed controls. Association statistics for the 4-variant panel were strong, with a P value of 3.50E-06 and high effect size (OR = 8.67). The population attributable fraction (PAF), or percentage of drug-induced PML cases that could be avoided with preventative genetic testing, is 9.4%.

**Table 5:** Clinical validity and utility of a 4-variant PML genetic risk test in drug-exposed cases vs. matched controls.

**Association statistics^a^**
Frequency in PML cases (12/110)^b^	10.9%
Frequency in matched controls (10/718)	1.4%
P value	3.50E-06
OR (95% CI)	8.7 (3.7–20.6)
**Clinical validity^c^**
Sensitivity	10.9%
Specificity	98.6%
PPV	19.5%
NPV	97.3%
**Clinical utility^c^**
PAF	9.4%
NNT	6
NNG	355

a Frequencies and statistics for drug-exposed PML cases and drug-exposed controls testing positive with the 4-variant PML genetic risk test: P values were calculated using Fisher's Exact Test; OR, odds ratio; CI, confidence interval.

b Details for the 12 genotype-positive PML cases are as follows: *C8B* variant 1-57409459-C-A (4 total), 4 natalizumab-treated MS patients; *FCN2* variant 9-137779251-G-A (5 total), 2 natalizumab-treated MS patients, 1 dimethyl fumarate-treated MS patient (natalizumab-naïve), 1 rituximab-treated B cell lymphoma patient, and 1 rituximab-treated Behcet's disease patient that also had immune thrombocytopenia; *LY9* variant 1-160769595-AG-A (1 total), 1 natalizumab-treated MS patient; *STXBP2* variant 19-7712287-G-C (2 total), 2 natalizumab-treated MS patients.

c Clinical validity and utility (also known as population impact) measures were calculated as described in Tonk et al. (51): PPV, positive predictive value; NPV, negative predictive value; PAF, population attributable fraction; NNT, number needed to treat; NNG, number needed to genotype. Values were calculated using a 3% adverse event frequency (PML incidence rate): JCV+ patients taking natalizumab and receiving at least 72 infusions (17).

In the total cohort of PML cases (*n* = 336), three of the four variants were found in both EUR and AFR cases (Table 6). All four variants were distributed across multiple primary disease subgroups, further supporting their association with PML rather than any one of the underlying disease groups (BC, HIV, MS, Other). In the drug-exposed PML cases (*n* = 110), three of the variants were found only in the MS subgroup (Table 6, footnote a), likely due to the high proportion of MS cases (Table 1, 86/110). However, the *FCN2* variant was found in three primary disease subgroups (BC, MS, Other) and in PML cases exposed to one of three different drugs (1 dimethyl fumarate case, 2 natalizumab cases, and 2 rituximab cases; see Supplementary Table 8). Taken together, these results suggest that the 4-variant PML risk genetic test could be used for advising on PML risk in general and for preventing iatrogenic PML cases.

**Table 6:** Distribution of genotype-positive PML cases^a^ across ethnicities and primary diseases.

**Gene symbol**	**dbSNP ID**	**Variant (GRCh37, hg19)**	**Primary ethnicity^b^**	**Primary disease^c^**
*STXBP2*	rs35490401	19-7712287-G-C	4 EUR	1 HIV, 2 MS, 1 Other
*LY9*	rs763811636	1-160769595-AG-A	1 EUR, 2 AFR	1 HIV, 1 MS, 1 Other
*C8B*	rs139498867	1-57409459-C-A	7 EUR, 2 AFR	1 BC, 4 HIV, 4 MS
*FCN2*	rs76267164	9-137779251-G-A	9 EUR, 1 AFR	2 BC, 4 HIV, 3 MS, 1 Other

a Results are shown for all PML cases (n = 336). Results for each of the 4 variants in the drug-exposed PML cases (n = 110): *STXBP2*, 2 MS; *LY9*, 1 MS; *C8B*, 4 MS; *FCN2*, 1 BC, 3 MS, 1 Other.

b Number of genotype-positive PML cases assigned to European (EUR) or African (AFR) ancestry (see Methods).

c Number of genotype-positive PML cases assigned to 1 of 4 primary disease subgroups (see Methods): BC, blood cancer; HIV, Human immunodeficiency virus infected; MS, multiple sclerosis; Other, various other diseases/conditions (see Methods).

## Discussion

### Four actionable risk variants identified from case-control analysis

With the addition of 152 PML cases to our previously studied 184 PML cases (45), we have now assembled the largest collection of PML DNA samples (*n* = 336) for studying germline genetics to identify variants associated with PML risk. One crucial improvement to our previous work is the assembly of drug-exposed matched controls (*n* = 718), defined as JCV+ MS patients who did not develop PML after being exposed to an immunosuppressant therapy with PML risk for ≥2 years. This cohort enabled us to conduct a targeted, case-control analysis on the previously identified set of 19 PML genetic risk variants. From this analysis we demonstrate the clinical validity and utility of four immune-linked, high effect size, rare variants for use in an iatrogenic PML risk genetic test in the following genes: *C8B, FCN2, STXBP2*, and *LY9* (Tables 2–5).

Individually, the four variants show strong associations in the drug-exposed cases vs. matched controls and gnomAD population controls (Table 2). Notably, the *LY9* and *STXBP2* variants were absent in the 718 drug-exposed controls. There was no association with MS for three of the variants (Table 4) and the fourth variant was not evaluated in the study, presumably due to its rarity (49). When combined as a single PML risk test, the top four variants show robust statistical associations, with a P value = 3.5E-06 and OR = 8.7 (Table 5). They were present in 10.9% of PML cases vs. only 1.4% of drug-exposed matched controls. As such, testing for these four variants could prevent a substantial number of patients from developing PML without deterring most patients from their treatment plan. Finally, each of the four variants appears to be individually predictive of PML risk, as no PML case or matched control had more than one of these variants. This is consistent with the hypothesis that rare, deleterious variants in immune-regulating genes confer risk of PML.

### Pharmacovigilance with a PML risk test is supported by clinical validity and utility measures

Clinical validity (sensitivity, specificity, PPV, NPV) refers to a test's ability to accurately predict a disorder while clinical utility (PAF, NNT, NNG), also referred to as population impact, measures its impact on the disorder (in this situation, PML cases prevented). See Tonk et al. (51) for further background information on pharmacogenetic test measures. The clinical impact of screening patients considering PML-linked drugs is shown in Table 5. The pharmacogenetic test measures shown are based on the results of this study and the rate of PML (3%) observed in JCV+ long duration natalizumab patients (17). One PML case would be prevented for every 355 patients genotyped (NNG). For every six patients (NNT) who carry one of these variants, one case of PML can be avoided. Additionally, the PAF of 9.4% suggests that nearly 10% of drug-exposed PML cases could be prevented. Taken together, preventative genotyping of patients considering treatment with a PML-linked drug would eliminate a significant portion of iatrogenic PML cases without deterring otherwise tolerant users (98.6% of the patient population who are not carriers of any of the top four variants) from starting or continuing treatment.

### Comparison to other clinically important genetic tests

**Figure 2:**
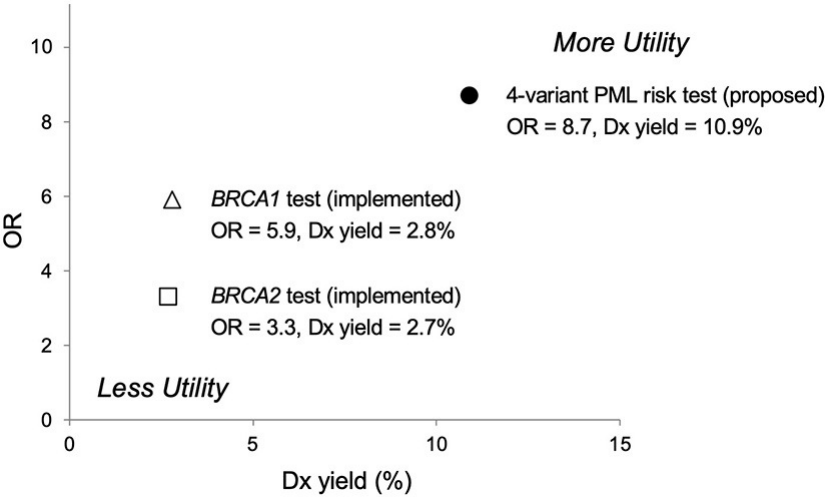
Predictive risk comparison to *BRCA* screening tests, odds ratio (OR) vs. diagnostic (Dx) yield. Results for a 4-variant PML risk test are shown in comparison to the *BRCA1*/*BRCA2* breast cancer risk prediction test. The proposed 4-variant PML risk test data point (●) is based on the total drug-exposed PML cases (Table 5). The *BRCA1* (Δ) and *BRCA2* (□) risk test data points are based on results for over 95,000 women reported in Kurian et al. (53). More Utility is defined as higher OR and higher Dx yield and Less Utility is defined as lower OR and lower Dx yield.

Comparisons to other clinically important genetic tests suggest that pre-treatment screening with our PML risk test would be appropriate and could reduce the occurrence of PML for any therapy with known or suspected PML risk. As shown in Figure 2, the results of a large study (95,961 patients) published in 2017 (53) reports lower OR values for the association of breast cancer with all known pathogenic variants in either *BRCA* gene (OR = 5.9 for *BRCA1*, OR = 3.3 for *BRCA2*) than the proposed 4-variant PML risk test (OR = 8.7). Moreover, this PML risk panel test was positive for 10.9% of PML cases in our study (Table 5), which is higher than the presence of *BRCA1* and *BRCA2* variants in breast cancer patients (2.8 and 2.7%, respectively) (53).

**Figure 3:**
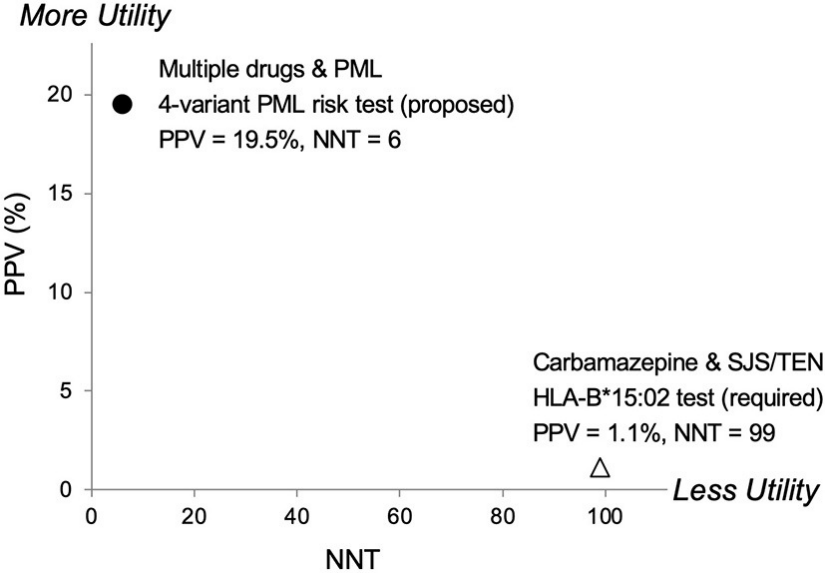
Predictive risk comparison to HLA-B*1502, positive predictive value (PPV) vs. number needed to treat (NNT). Results for a 4-variant PML risk test are shown in comparison to the HLA-B*15:02 test that is required in Asian populations before administering carbamazepine (CBZ). CBZ is a cause of the serious adverse event Stevens-Johnson Syndrome (SJS) and Toxic Epidermal Necrolysis (TEN). The proposed 4-variant PML risk test data point (●) is based on the total drug-exposed PML cases (Table 5) and a PML incidence rate of 3% (17). The HLA-B*15:02 SJS/TEN risk test data point (Δ) is based on results reported in Shi et al. (54). More Utility is defined as higher PPV and lower NNT and Less Utility is defined as lower PPV and higher NNT.

Another relevant comparison is carbamazepine, an anticonvulsant drug. The FDA added a Boxed Warning to its prescribing information requiring pre-treatment genetic testing in certain populations for HLA-B^*^15:02 due to Stevens-Johnson syndrome/Toxic Epidermal Necrolysis (SJS/TEN) risks. In Figure 3, a comparison of pharmacogenetic testing measures (PPV vs. NNT) as indicators of clinical utility shows the proposed PML risk panel test has the potential to provide greater utility than the currently recommended test for carbamazepine's HLA-B^*^15:02 association with SJS/TEN (55). Also note that mortalities associated with SJS and TEN are estimated at 1–5% and 25–35%, respectively (56). Whereas PML-associated mortality is higher, reported as 23–65% (1, 15).

### The top four variants are predicted to be pathogenic and have strong biological connections

In addition to being supported by strong statistical, clinical validity, and clinical utility measures, the four variants proposed for inclusion in the PML risk panel are predicted to be deleterious (Table 3) and their rarity further supports that they are pathogenic. All of the genes in which the four variants are located are linked to the immune system's viral defense mechanisms. Two genes (*C8B* and *FCN2*) are part of the complement system (lectin and terminal pathways) (57–60). The other two genes (*LY9* and *STXBP2*) cause or are linked to hemophagocytic lymphohistiocytosis (HLH) disorders, including macrophage activation syndrome (MAS) (61–65). Two genes (*C8B* and *STXBP2*) with PML risk variants are among the 437 genes designated by the International Union of Immunological Societies (IUIS) to cause inborn errors of immunity (43, 44), thereby supporting our original hypothesis (25) of host genetics as an additonal risk factor for development of PML.

### Study limitations

A few areas of limitation are noted. Of the 336 PML cases, 49 had insufficient information to confirm them as Definite PML (47). Since PML is rare, assembling patient cohorts for research studies is very challenging and several of our cases were documented before consensus diagnostic criteria were implemented. Therefore, we decided to include both Definite and Probable PML cases in our study. Of note, Probable cases were almost entirely collected from PML centers of excellence, increasing the likelihood that they are in fact genuine PML cases.

The ethnic diversity for our PML cases is somewhat limited (Table 1 and Methods). While 11/336 (3%) PML cases assigned as EUR ancestry to simplify association analyses (see Methods) formally belonged to another majority ancestry, our study is lacking in predominantly East Asian, South Asian, and Latino/Admixed American ancestries. For example, studying germline genetics in East Asians may help to explain why PML incidence rates are about 8-fold higher in Japan for fingolimod-treated MS patients compared to the US and worldwide rates (66, 67). Presently, a higher rate of JCV seropositivity (2–5) and HLA-DRB1 alleles have been suggested as potential factors for this higher rate of PML development in fingolimod-treated Japanese MS patients (68).

We note that 95/336 (28%) PML cases had mixed ancestry (i.e., at least one other ancestry present at > 5%) and all four of our top variants are globally rare for all gnomAD ethnicities (allele frequency range 0.000072 to 0.004331). Furthermore, variant associations were significant whether analyzed by primary ethnicity (EUR or AFR) or using all ethnicities (All pooled analysis). While it is important to continue to study PML cases in underrepresented ancestries, we believe the global rarity of these variants—combined with the strength of the associations observed here—obviates the need to assess the variants in specific populations and enables the use of our PML risk test in the general population.

For iatrogenic PML cases, drug-specific association results may be informative but in our present work, this was only possible for the subgroup of PML cases and matched controls that were exposed to natalizumab (86 cases vs. 604 controls); see Supplementary Tables 6, 9. The next largest drug-specific subgroup for the drugs listed in Table 1 was rituximab (13 cases vs. 25 controls). However, given the consistent results (Supplementary Table 9) for our 4-variant PML genetic risk test among natalizumab-exposed and all drug-exposed cases and controls (110 cases vs. 718 controls), we would not expect dramatic differences across drug-specific groups. Furthermore, one of our PML risk variants (in the *FCN2* gene) was found in PML cases (see Table 5, footnote b) with three different underlying diseases (MS, B cell lymphoma, and Behcet's disease with immune thrombocytopenia), but also representing exposure to three different PML-linked drugs (dimethyl fumarate, natalizumab, and rituximab).

Finally, using matched controls for other underlying disorders besides MS (e.g., leukemia/lymphoma, other autoimmune conditions, or HIV-infected patients who did not develop PML) may provide additional support to the use of our proposed PML risk test in other clinical settings. Beyond the practical limitations of performing PML risk case-control studies for other underlying disorders and drug exposures, these results suggest it is likely unnecessary. All four top variants were found in PML cases representing at least three of four primary disease subgroups, with the *FCN2* variant found in all four subgroups (Table 6). This is consistent with the understanding that PML is the same clinical entity regardless of the patient's underlying disorder (1) and supports the use of our test in all patients considering the use of PML-linked therapies.

## Conclusion

Identification of patients at risk of PML is an important area of unmet need given the growing number of PML-linked immunosuppressive therapies. Building on our previous work (45), this study represents what we believe to be the first case-control analysis of germline genetic variants that confer risk of PML. The association of PML risk with damaging variants in the immune-linked genes *C8B, FCN2, LY9*, and *STXBP2* is confirmed, with two variants being completely absent in the drug-exposed controls. High OR values and statistical significance support the use of this information when assessing patient risk of PML. The underlying genetic immunodeficiency conditions linked to these variants predispose carriers to uncontrolled JCV virus reactivation (i.e. PML), a serious infection. Simple, low-cost genetic screening in patients considering drugs with known or suspected PML risk will prevent future cases. Due to the seriousness of a PML diagnosis—particularly because it often leads to life-threatening outcomes (69) and the lack of treatment options once it develops—it would seem unethical not to test individuals considering immunosuppressive therapies with PML risk for our top four variants, and advising those with a positive result to consider an alternative therapy or treatment strategy.

## Data availability statement

The original contributions presented in the study are publicly available. This data can be found here: National Center for Biotechnology Information (NCBI) ClinVar, https://www.ncbi.nlm.nih.gov/clinvar/, SCV002572501.1, SCV002572502.1, SCV002572503.1, and SCV002572504.1.

## Ethics statement

The studies involving human participants were reviewed and approved by IRB protocols from the following institutions: Accelerated Cure Project, Comitato Etico Provinciale of Brescia (LI), Beth Israel Deaconess Medical Center (IK), Icahn School of Medicine at Mount Sinai (BioMe Biobank), NINDS/NIH (EM and IC), Paris-Sud/INSERM (YT), University of California San Francisco (JO), University of Münster (NS and HW), Université Toulouse (DB, GM-B, and RL), and Vanderbilt University (BioVU Biobank). The patients provided their written informed consent to participate in this study.

## Author contributions

EH, ES, and PE: conception and design of the study. EH, SJ, and DR: laboratory experiments. EH, ES, SJ, CB, TR, and PE: data analysis and interpretation. YT, HH-C, RL, DB, GM-B, HW, NS, IC, MM, LI, RC, JO, JG, BS, IK, BH, and EM: provision of study materials and clinical information for patients. EH, ES, CC, and PE: wrote the manuscript. All authors revised/approved the manuscript.
