# Supplementary material for: Progressive multifocal leukoencephalopathy genetic risk variants for pharmacovigilance of immunosuppressant therapies
Source: Front Neurol. 2022 Dec 14;13:1016377. doi: 10.3389/fneur.2022.1016377 (PMC9795231; doi:10.3389/fneur.2022.1016377)
Supplement: Supplementary file 1 [file Data_Sheet_1.pdf]

Supplementary Material

Data Supplement

Additional Genetic Validation Results

Overview of the Study and New Cohorts

A set of 19 previously reported (1) germline genetic risk variants associated with progressive multifocal leukoencephalopathy (PML) were further validated in additional PML cases. Association analyses were performed using population and matched controls. For the publicly available population controls, we used the large multi-ethnic Genome Aggregation Database (gnomAD) (2). New PML cases and matched controls were assembled and genotyped for the 19 variants and pooled association analyses were also performed that included whole exome sequencing (WES) data previously reported for 184 PML cases (1). Based primarily upon an analysis of 110 drug-exposed cases against 718 drug-exposed matched controls but also informed by various analyses of cases and population controls, four variants were selected for a PML genetic risk test. See **Figure 1** (main text) for the workflow.

Like our previously studied PML cases (n = 184) (1), newly assembled Cohort 3 (n = 152) comprises patients with a primary disease across one of four subgroups (BC, HIV, MS, or Other), see **Supplementary Table 1** and Methods for details. Pooled analyses used a total of 336 PML cases (prior study plus Cohort 3) and 110 drug-exposed cases (patients exposed to at least one PML-linked drug who developed PML). Not surprisingly, HIV patients with PML represent the largest number of total cases in our collection (156/336) since at least 50% of PML occurs in this patient group (3). Another crucial component of our current genetic validation study is the assembly of drug-exposed matched controls (n = 718, see **Table 1** in main text), defined as multiple sclerosis (MS) patients seropositive for the JC virus (JCV+) who did not develop PML after being exposed to a PML-linked immunosuppressant therapy for ≥ 2 years. These matched controls are representative of one of the intended patient groups for the PML risk test (i.e., MS patients in need of an immunosuppressant therapy). This enabled us to compare association analyses for our original set of 19 PML genetic risk variants (**Supplementary Table 2**) using two sets of controls, gnomAD population controls (**Supplementary Tables 3-5**) and drug-exposed controls (**Supplementary Table 5**). Results for a natalizumab-exposed subset of the drug-exposed data are also reported (**Supplementary Table 6** and **Supplementary Table 9**).

Summary of Candidate PML Risk Variants

Candidate PML risk variants genotyped in PML Cohort 3 are those reported in Eis et al. 2020 (1) and are summarized, along with their predicted functional impact, in **Supplementary Table 2.** Nineteen variants in 17 genes (*IGLL1* and *PLCG2* each had two variants) are listed and the consequence for 17 of 19 variants is a change in amino acid (missense), but two are predicted to cause protein loss of function (pLOF). In addition to Polyphen and SIFT, we updated the *in silico* functional prediction for each variant to include CADD scores. A majority (16 of 19) were found to be deleterious by one or more of the three methods.

Of the 17 genes, 11 are designated by the International Union of Immunological Societies (IUIS) to be genes that cause inborn errors of immunity. The number of disease genes identified by the IUIS

continues to grow. In our original study (1), we assembled a list of 337 IUIS genes and this list has now expanded to 437 based on recent updates (4, 5). Of our 17 previously reported PML risk candidate genes, *DNASE1L3* is now also considered an IUIS gene (**Supplementary Table 2**).

Association Analysis of Candidate PML Risk Variants in All PML Cases Using Population Controls

Association analyses were initially performed using gnomAD 3.1 population control data, which are similar to the previously used gnomAD 2.1 population controls (1). The main difference between these versions is that 3.1 includes only WGS data (~76,000 genomes corresponding to all ethnicities) and 2.1 includes WGS and WES data (~141,000 genomes corresponding to all ethnicities: ~126,000 WES and ~16,000 WGS).

Prior analyses (1) were performed on an ethnic-specific basis using the gnomAD 2.1 data (EUR PML cases vs. NFE subjects and AFR PML cases vs. AFR subjects). However, we noted that 10 of 19 variants were found in both EUR and AFR ancestry PML cases. Therefore, in the current analyses (**Supplementary Table 3**), we simply used all subjects in gnomAD 3.1, regardless of ethnicity. Supporting data for this approach are reported in **Supplementary Table 4** (EUR, AFR, and EUR+AFR analyses) as a comparison to **Supplementary Table 3** data (pooled Cohorts 1-3 vs. gnomAD 3.1 All ethnicities) and show that this is a reasonable approach since the majority of the variants are rare in all ethnicities (allele frequencies are < 0.008). One exception was an allele frequency of 0.019 reported for 6-30673359-T-G, which is based on the allele frequency data for a nearby SNV (6-30671312-A-C, rs28994874) known to be in linkage disequilibrium with 6-30673359-T-G. We note that since our original study (1), it was determined that 6-30673359-T-G lies within a region whose mappability results in an artificially low allele number (thereby skewing the association statistics). Importantly, a pan-ethnic approach simplifies the interpretation of a genetic test (i.e., the variants are statistically significant in all ethnicities) and precludes the need to perform ancestry testing on test recipients or assume that their reported ethnicity is correct. For example, **Supplementary Table 1** shows that 60% (33/55) of AFR PML cases and 22% (62/281) of EUR PML cases also had another ethnicity present at >5%.

As noted above, in **Supplementary Table 3** PML cases were analyzed using the pooled collection of Cohorts 1-3. Given the rarity of PML, a case vs. control study design typical of GWAS (thousands of cases vs. thousands of controls) was not possible. Pharmacogenomics researchers have recognized this issue with efficacy and safety studies involving rare variants and therefore developed variant annotation scoring metrics for small cohorts (e.g., 50-500 cases) as well as giving additional weight to hypothesis-driven studies (6), which was the basis of our study design (1, 7). The pooled analysis revealed statistically significant results (p-value ≤ 0.05) for 13 of 19 variants.

Association Analysis of Candidate PML Risk Variants in Drug-Exposed PML Cases Using Population Controls and Matched Controls

PML occurs in the context of a range of disorders (3), most notably in HIV (~50%), but iatrogenic causes of PML (i.e., PML occurring as a serious adverse event from a PML-linked drug) represent an important proportion of cases (e.g., ~10% are patients with hematological malignancies, a subset of which are treated with PML-linked drugs, and ~5% are MS patients on disease-modifying therapies). Iatrogenic cases are the focus of our host genetics research since these are potentially preventable with implementation of a PML risk genetic test. Therefore, we performed statistical analyses of a drug-exposed subgroup (n = 110, **Supplementary Table 5**) of our total set of PML cases (n = 336, **Supplementary Table 3**). The drug-exposed subgroup was analyzed using gnomAD 3.1 population controls and 718 drug-exposed matched controls.

In the drug-exposed subgroup of 110 PML cases, exposures included seven drugs (**Supplementary Table 1**), all of which have PML listed in their prescribing information (in a Boxed Warning and/or in the Warnings and Precautions section) except for glatiramer acetate. Drug exposures in the drug-exposed matched controls included five drugs (**Table 1**, main text), four (dimethyl fumarate, fingolimod, natalizumab, and rituximab) of which overlap with drug exposures in the PML cases.

Association analyses using the gnomAD 3.1 population controls (**Supplementary Table 5**), shows that 5 of 19 candidate PML risk variants were significant (p-value ≤ 0.05). Encouragingly, with the drug-exposed controls, the same variants were significant with the exception of the *LY9* and *MCM5* variants. In PML cases, 1/110 was positive for the *LY9* variant and it was not found in the drug-exposed matched controls. Despite not having a significant p-value (1.33E-01) against the drug-exposed matched controls, the effect size for the *LY9* variant is high (OR = 19.60) and thus merited further consideration as a top PML risk variant. The *MCM5* variant, which did not have a significant p-value (2.84E-01) and a lower OR (1.89) than was found with the gnomAD 3.1 controls (p-value = 1.58E-02, OR = 4.33), will require further validation (e.g., genotyping in additional PML cases and drug-exposed matched controls) before consideration as a top PML risk variant.

Given the large number of natalizumab-exposed PML cases (n = 86) in our study, we also reported the association results for this subgroup (**Supplementary Table 6**) and found that the results were comparable to the full set of drug-exposed PML cases (n = 110).

No Association for Candidate PML Risk Variants with MS

Since MS patients are one of the intended patient groups for a PML risk genetic test, we checked if any of our candidate PML risk variants were also associated with MS. Previously reported MS GWAS data from a large international study were used (8). This MS study used an exome chip (Illumina) containing 137,007 genome-wide common (12%) and rare (88%) variants to identify MS-associated loci in 32,367 MS cases vs. 36,012 healthy controls.

Association statistics in MS cases vs. healthy controls were available for 12 of 19 candidate PML risk variants (**Supplementary Table 7**). The p-values for the 12 variants ranged from 0.02654 to 0.93770, none of which are significant since they were not corrected for multiple testing. Effect sizes for the 12 variants also did not support an association with MS as the OR values ranged from 0.9420 to 1.0085. Notably, 3 of 4 candidate PML risk variants (in genes *C8B*, *FCN2*, and *STXBP2*) in the drug-exposed subgroup (**Supplementary Table 5)** under consideration for our PML risk test are not associated with MS. The *LY9* variant was not present on the exome chip, presumably because it is very rare (allele frequency in gnomAD 3.1 is 0.000072, see **Supplementary Table 3**). Given the large size of this international MS study, we concluded that our top candidate PML risk variants were not associated with MS and could therefore be used in a PML risk genetic test.

Variant Distribution

Supplementary Table 8 details variant distribution across ethnicities, primary diseases, and drug exposures. While only 55/336 (16%) PML cases had AFR as their primary ethnicity (**Supplementary Table 1**), 10 of 19 candidate PML risk variants were present in both AFR and EUR PML cases and 16 of 19 variants (**Supplementary Table 8)** had a secondary ethnicity (>5% of one more other ethnicities besides the primary ethnicity). This underscores (along with association results reported in **Supplementary Table 3** and **Supplementary Table 4**) that most of our candidate PML risk variants can be tested in patients regardless of their ethnic background.

Of the four primary disease subgroups (BC, HIV, MS, and Other; see **Supplementary Table 1** and Methods), 13 of 19 variants were present in two or more subgroups, 9 of 19 variants were present in three or more subgroups, and 3 of 19 variants were present in all four subgroups (**Supplementary Table 8**). This provides strong supporting evidence that our variants are reporting on PML risk, rather than being linked to the pathology of the underlying disease in the PML cases. Finally, while drug exposure information was only available for 110/180 (61%) PML cases (HIV PML cases are not included in this subgroup), 10 of 19 variants were found in PML cases exposed to at least one drug linked to PML and two variants were found in PML cases exposed to either two (*MCM5*; natalizumab, rituximab) or three (*FCN2*; dimethyl fumarate, natalizumab, rituximab) different PML-linked drugs. This suggests that a subset of our candidate PML risk variants can be used in a PML risk genetic test for multiple PML-linked drugs.

Top Four Variants Selected for a PML Risk Test

Based on the assessment of pooled association analyses for all PML cases (n = 336, **Supplementary Table 3**) and the drug-exposed PML cases (n = 110, **Supplementary Table 5**), four variants were selected for use in a 4-variant panel test for PML risk (**Supplementary Table 9**). Individually, the four variants each had robust associations in the gnomAD population controls for all PML cases (p-values 2.96E-05 to 1.46E-02) and in the drug-exposed subset (p-values 9.56E-04 to 3.73E-02). In the drug-exposed matched control analyses, reasonable associations were found for all four variants (p-values 9.03E-03 to 1.33E-01) considering that the matched controls cohort size (n = 718) is ~100-fold smaller than the gnomAD population controls cohort size (~76,000 subjects). Effect sizes were high for all analyses: 3.15 to 62.28 for all PML cases vs. gnomAD population controls; 4.32 to 63.42 for drug-exposed PML cases vs. gnomAD population controls; 5.65 to 33.11 for drug-exposed PML cases vs. drug-exposed matched controls.

Composite association analyses (**Supplementary Table 9**) for the 4-variant panel test were robust for all comparisons: 336 PML cases vs. 76,071 gnomAD controls (p-value = 1.36E-09, OR = 4.51), 110 drug-exposed PML cases vs. 76,071 gnomAD controls (9.60E-07, OR = 6.58), and 110 drug-exposed PML cases vs. 718 drug-exposed controls (3.50E-06, OR = 8.67). We note that no PML cases or drug-exposed matched controls had more than one of the top four variants. In other words, each of these variants is predictive of PML risk and this is reflected in the composite frequency of the variants in PML cases (e.g., in the 110 drug-exposed PML cases, 10.9% had 1 of 4 variants, whereas individually the frequencies were 0.9-4.5%).

Using data from **Supplementary Table 6**, we also report association results for the natalizumab-exposed subgroup for each of the top four variants and the 4-variant panel (**Supplementary Table 9**). Composite association results for the 4-variant panel were essentially the same for drug-exposed versus natalizumab-exposed subgroups (using matched controls, p-values of 3.50E-06 and 2.68E-05 and ORs of 8.67 and 9.97).

Our proposed PML risk panel is pan-ethnic. Since 20% of EUR PML cases and 60% of AFR PML cases (**Supplementary Table 1**) had mixed ethnicity (one or more Other ethnicity > 5%), we confirmed that the top four variants were globally rare (**Supplementary Table 3**, gnomAD 3.1 allele frequency range 0.000072 to 0.004331) and had reasonable associations if analyzed using only the primary ethnicity (EUR or AFR, **Supplementary Table 4**) as compared to using all ethnicities (All pooled analysis, **Supplementary Table 3**). In contrast, some genetic variants linked to severe adverse events that are on drug labels with boxed warnings and mandated testing are present at highly variable frequencies among ethnic groups (e.g., 0 to 22% for HLA-B*1502), which can make it more difficult to interpret the test results as well as determine the cost-effectiveness of pharmacovigilance (9). Clinical validity and utility

of our 4-variant PML risk test is compelling (**Table 5**, main text), including in comparison to other genetic risk tests (**Figures 2** and **3**, main text).

As more PML cases are assessed, we anticipate adding additional PML risk variants to the genotyping panel. For example, while the *MCM5* variant (22-35806756-G-A, rs2230933) achieved significance when analyzed against the gnomAD population controls (n = ~76,000), it was not significant using the drug-exposed matched controls (n = 718) (**Supplementary Table 5**). For now, we chose to conservatively include only variants (**Tables 2-6**, main text) that were strongly associated with PML in both sets of controls (population and matched).

References

1. Eis PS, Bruno CD, Richmond TA, Koralnik IJ, Hanson BA, Major EO, et al. Germline Genetic Risk Variants for Progressive Multifocal Leukoencephalopathy. Front Neurol. 2020;11:186.

2. Karczewski KJ, Francioli LC, Tiao G, Cummings BB, Alfoldi J, Wang Q, et al. The mutational constraint spectrum quantified from variation in 141,456 humans. Nature. 2020;581(7809):434-43.

3. Cortese I, Reich DS, Nath A. Progressive multifocal leukoencephalopathy and the spectrum of JC virus-related disease. Nat Rev Neurol. 2021;17(1):37-51.

4. Tangye SG, Al-Herz W, Bousfiha A, Chatila T, Cunningham-Rundles C, Etzioni A, et al. Human Inborn Errors of Immunity: 2019 Update on the Classification from the International Union of Immunological Societies Expert Committee. J Clin Immunol. 2020;40(1):24-64.

5. Tangye SG, Al-Herz W, Bousfiha A, Cunningham-Rundles C, Franco JL, Holland SM, et al. The Ever-Increasing Array of Novel Inborn Errors of Immunity: an Interim Update by the IUIS Committee. J Clin Immunol. 2021;41(3):666-79.

6. Whirl-Carrillo M, Huddart R, Gong L, Sangkuhl K, Thorn CF, Whaley R, et al. An Evidence-Based Framework for Evaluating Pharmacogenomics Knowledge for Personalized Medicine. Clin Pharmacol Ther. 2021;110(3):563-72.

7. Hatchwell E. Is there a (host) genetic predisposition to progressive multifocal leukoencephalopathy? Front Immunol. 2015;6:216.

8. International Multiple Sclerosis Genetics Consortium. Electronic address ccye, International Multiple Sclerosis Genetics C. Low-Frequency and Rare-Coding Variation Contributes to Multiple Sclerosis Risk. Cell. 2018;175(6):1679-87 e7.

9. Zhou Y, Krebs K, Milani L, Lauschke VM. Global Frequencies of Clinically Important HLA Alleles and Their Implications For the Cost-Effectiveness of Preemptive Pharmacogenetic Testing. Clin Pharmacol Ther. 2021;109(1):160-74.

SUPPLEMENTARY TABLE 1 | Summary of PML cases by cohort: demographics, primary disease group, and MS drug exposure**.**

	PML Cases[a]		
	**Cohorts 1+2**	**Cohort 3**	**Total**
**Subjects**	184	152	336
**Sex**			
Female	66	86	152
Male	118	66	184
**Primary ethnicity[b]**			
AFR	49	6	55
AFR with Other ethnicity > 5%	29 (59%)	4 (67%)	33 (60%)
EUR	135	146	281
EUR with Other ethnicity > 5%	42 (31%)	20 (14%)	62 (22%)
**Primary disease[c]**			
BC	23	18	41
HIV	128	28	156
MS	15	79	94
Other	18	27	45
**MS drug exposure[d]**			
Alemtuzumab	0	1	1
Dimethyl fumarate	1	2	3
Fingolimod	0	1	1
Glatiramer acetate	0	1	1
Mycophenolate mofetil	0	2	2
Natalizumab	12	74	86
Rituximab	1	12	13
Unknown[e]	2	2	4
Total Drug-exposed subsets (non-redundant)	16	94	110

[a] Cohorts 1+2 were previously reported (1); Cohort 3 contains newly recruited PML cases.

[b] A primary ethnicity was assigned as AFR or EUR (see Methods) for statisical analyses. An Other ethnicity was annotated if > 5% was found, with the number noted and % of total number in parentheses.

[c] Primary Disease: BC = blood cancer, HIV = human immunodeficiency virus infected, MS = multiple sclerosis, Other = various; see Methods for list of diseases under the BC and Other subgroups.

[d] All of the drugs (either indicated or used off-label for MS) have PML listed in the prescribing information (Boxed Warning and/or Warnings and Precautions) with the exception of glatiramer acetate. Drug exposure times were unavailable for PML cases but are ≥ 2 years for controls (a subset had ≥ 2 years for two or more MS drugs). Of the 110 drug-exposed PML cases, four had multiple reported drug exposures: 1 glatiramer acetate (also exposed to interferon beta-1a) and 3 rituximab (also exposed to bendamustine, cyclophosphamide-fludarabine, or cyclosporine-methotrexate-mycophenolate mofetil-steroids-tacrolimus, respectively).

[e] Four PML cases had unknown drug exposures but were assumed to be drug-exposed since all were MS patients, a patient group that is not known to develop PML in the absence of treatment with a disease-modifying therapy.

SUPPLEMENTARY TABLE 2 | Candidate PML risk variant information.[a]

Gene symbol	IUIS-437[b]	dbSNP ID	Variant (GRCh37, hg19)	Variant (GRCh38, hg38)	Consequence[c]	Protein impact	gnomAD 3.1 *in silico* predictions: Polyphen	SIFT	CADD[e]
AIRE	yes	rs148012328	21-45708278-G-A	21-44288395-G-A	missense	p.Gly197Arg	probably _damaging	tolerated	22.7
C8B	**yes**	**rs139498867**	**1-57409459-C-A**	**1-56943786-C-A**	**missense**	**p.Asp382Tyr**	**possibly _damaging**	**deleterious**	**23.1**
CFHR2	yes	rs148175483	1-196918605-A-G	1-196949475-A-G	missense	p.Lys27Glu	probably _damaging	tolerated	0.745
DNASE1L3	yes	rs12491947	3-58191230-G-T	3-58205503-G-T	missense	p.Asn96Lys	benign	tolerated	0.787
FCN2	**no**	**rs76267164**	**9-137779251-G-A**	**9-134887405-G-A**	**missense**	**p.Arg311Gln**	**probably _damaging**	**deleterious**	**24.0**
GFI1	yes	rs149914857	1-92946625-G-C	1-92481068-G-C	missense	p.Pro107Ala	benign	deleterious	22.8
IFIH1	yes	rs35337543	2-163136505-C-G	2-162279995-C-G	splice donor (pLOF)	n/a	n/a[d]	n/a[d]	32.0
IGLL1	yes	rs1064421	22-23915583-T-C	22-23573396-T-C	missense	p.Asn171Ser	possibly _damaging	tolerated	8.46
IGLL1	yes	rs143780139	22-23915745-G-A	22-23573558-G-A	missense	p.Thr117Ile	benign	tolerated	10.8
LIG1	yes	rs3730947	19-48643270-C-T	19-48140013-C-T	missense	p.Val349Met	possibly _damaging	deleterious	21.5
LY9	**no**	**rs763811636**	**1-160769595-AG-A**	**1-160799805-AG-A**	**frameshift (pLOF)**	**p.Gly61ValfsTer3**	**n/a[d]**	**n/a[d]**	**22.8**
MCM5	no	rs2230933	22-35806756-G-A	22-35410763-G-A	missense	p.Val258Ile	possibly _damaging	tolerated	24.4
MDC1	no	rs143258964	6-30673359-T-G	6-30705582-T-G	missense	p.Thr1201Pro	benign	tolerated	0.012
NQO2	no	rs148024596	6-3015818-G-A	6-3015584-G-A	missense	p.Val120Met	probably _damaging	deleterious	27.7
PKHD1	no	rs199589074	6-51798908-C-T	6-51934110-C-T	missense	p.Gly2041Ser	probably _damaging	deleterious	33.0
PLCG2	yes	rs187956469	16-81939089-T-C	16-81905484-T-C	missense	p.Tyr482His	**possibly _damaging**	tolerated	24.1
PLCG2	yes	rs75472618	16-81942175-A-G	16-81908570-A-G	missense	p.Asn571Ser	benign	deleterious	22.7
STXBP2	**yes**	**rs35490401**	**19-7712287-G-C**	**19-7647401-G-C**	**missense**	**p.Arg529Pro**	**probably _damaging**	**deleterious**	**26.0**
TCIRG1	yes	rs75596506	11-67818269-G-A	11-68050802-G-A	missense	p.Ala826Thr	benign	tolerated	4.92

[a] Candidate PML risk variants were previously reported (1); entries in **bold** are also reported in Tables 2-6 (see main text). Gray-shading denotes severity of functional predictions: no shading = low impact, light gray = moderate impact, dark gray = high impact.

[b] IUIS genes that cause inborn errors of immunity now total 437 (4, 5).

[c] Missense variants are amino acid substitutions; for a subset of variants, pLOF = protein loss-of-function.

[d] Polyphen and SIFT are prediction methods for missense variants and are not applicable (n/a) to other types of variants (e.g., the *IFIH1* splice donor and *LY9* frameshift).

[e] CADD scores > 20 are the highest category of deleteriousness in gnomAD 3.1 annotation.

SUPPLEMENTARY TABLE 3 | Statistical analysis of candidate PML risk variants: PML Cohorts 1+2, Cohort 3, and Cohorts 1-3 (pooled PML cases) vs. gnomAD 3.1 population controls.[a]

				Cohorts 1+2			Cohort 3			Cohorts 1-3 (pooled)			
**Gene symbol**	**Variant (GRCh37, hg19)**	**gnomAD 3.1 AF[b]**	**gnomAD 3.1 subjects[b]**	**PML cases**	**p-value**	**OR**	**PML cases**	**p-value**	**OR**	**PML cases**	**p-value**	**OR**	**OR 95% CI**
AIRE	21-45708278-G-A	0.000237	36/76,111	2/184	3.84E-03	23.22	0/152	--	--	2/336	1.22E-02	12.65	3.03 - 52.61
C8B	**1-57409459-C-A**	**0.004331**	**659/76,081**	**5/184**	**2.31E-02**	**3.20**	**4/152**	**4.44E-02**	**3.09**	**9/336**	**3.08E-03**	**3.15**	**1.61 - 6.12**
CFHR2	1-196918605-A-G	0.000920	139/76,102	3/184	5.04E-03	9.06	0/152	--	--	3/336	2.51E-02	4.92	1.56 - 15.48
DNASE1L3	3-58191230-G-T	0.007575	1,134/76,109	2/184	1.00E+00	0.73	3/152	4.97E-01	1.33	5/336	1.00E+00	1.00	0.41 - 2.41
FCN2	**9-137779251-G-A**	**0.003393**	**511/76,050**	**7/184**	**2.88E-04**	**5.85**	**3/152**	**8.42E-02**	**2.98**	**10/336**	**1.23E-04**	**4.53**	**2.40 - 8.53**
GFI1	1-92946625-G-C	0.002595	393/76,104	4/184	1.61E-02	4.28	0/152	--	--	4/336	9.91E-02	2.32	0.86 - 6.23
IFIH1	2-163136505-C-G	0.007249	1,097/76,008	11/184	9.05E-05	4.34	2/152	1.00E+00	0.91	13/336	1.47E-03	2.75	1.73 - 5.07
IGLL1	22-23915583-T-C	0.007227	1,095/76,031	5/184	1.98E-01	1.91	0/152	--	--	5/336	8.18E-01	1.03	0.43 - 2.50
IGLL1	22-23915745-G-A	0.001945	296/76,092	4/184	6.23E-03	5.69	0/152	--	--	4/336	4.47E-02	3.08	1.14 - 8.30
LIG1	19-48643270-C-T	0.002772	421/76,114	3/184	8.38E-02	2.98	0/152	--	--	3/336	4.40E-01	1.62	0.52 - 5.05
LY9	**1-160769595-AG-A**	**0.000072**	**11/76,054**	**2/184**	**4.44E-04**	**75.97**	**1/152**	**2.37E-02**	**45.78**	**3/336**	**2.96E-05**	**62.28**	**17.24 - 223.58**
MCM5	22-35806756-G-A	0.004359	657/76,043	7/184	1.24E-03	4.54	4/152	4.41E-02	3.10	11/336	2.15E-04	3.88	2.11 - 7.10
MDC1	6-30673359-T-G[c]	0.019090	2,864/76,001	9/184	4.33E-01	1.31	7/152	5.21E-01	1.23	16/336	3.15E-01	1.28	0.77 - 2.11
NQO2	6-3015818-G-A	0.000631	96/76,105	3/184	1.83E-03	13.12	0/152	--	--	3/336	9.67E-03	7.13	2.24 - 22.55
PKHD1	6-51798908-C-T	0.000204	31/76,115	3/184	7.81E-05	40.68	0/152	--	--	3/336	4.55E-04	22.11	6.71 - 72.46
PLCG2	16-81939089-T-C	0.003364	511/76,104	5/184	8.61E-03	4.13	1/152	1.00E+00	0.98	6/336	2.77E-02	2.69	1.19 - 6.04
PLCG2	16-81942175-A-G	0.007856	1,187/76,054	11/184	1.77E-04	4.01	2/152	1.00E+00	0.84	13/336	2.85E-03	2.54	1.45 - 4.42
STXBP2	**19-7712287-G-C**	**0.001367**	**208/76,099**	**4/184**	**1.81E-03**	**8.11**	**0/151**	**--**	**--**	**4/335**	**1.46E-02**	**4.41**	**1.62 - 11.89**
TCIRG1	11-67818269-G-A	0.007104	1,072/76,089	7/184	1.64E-02	2.77	0/152	--	--	7/336	2.48E-01	1.49	0.70 - 3.15

[a] Candidate PML risk variants were previously reported (1), see Methods; entries in **bold** are also reported in Tables 2-6 (see main text). p-values were calculated using Fisher's Exact Test, OR = Odds Ratio, CI = Confidence Interval.

[b] All ethnicities in gnomAD 3.1 population controls were used (see Methods).

[c] Since the previous report (1), it was determined that 6-30673359-T-G lies within a region whose mappability results in an artificially low allele number for this variant. Hence, a nearby variant, known to be in linkage disequilibrium with 6-30673359-T-G, was used instead: 6-30671312-A-C (GRCh37), rs28994874.

SUPPLEMENTARY TABLE 4 | Statistical analysis of candidate PML risk variants: PML Cohorts 1-3 (pooled PML cases) vs. gnomAD 3.1 population controls by ethnicity (EUR, AFR, EUR+AFR).[a]

		EUR				AFR				EUR+AFR			
**Gene symbol**	**Variant (GRCh37, hg19)**	**PML cases**	**gnomAD subjects**	**p-value**	**OR**	**PML cases**	**gnomAD subjects**	**p-value**	**OR**	**PML cases**	**gnomAD subjects**	**p-value**	**OR**
AIRE	21-45708278-G-A	0/281	0/34,018	--	--	2/55	34/20,724	4.09E-03	22.96	2/336	34/54,742	2.04E-02	9.64
C8B	**1-57409459-C-A**	**7/281**	**466/34,021**	**1.16E-01**	**1.84**	**2/55**	**56/20,718**	**1.03E-02**	**13.92**	**9/336**	**522/54,739**	**5.73E-03**	**2.86**
CFHR2	1-196918605-A-G	0/281	0/34,016	--	--	3/55	129/20,727	5.16E-03	9.21	3/336	129/54,743	4.74E-02	3.81
DNASE1L3	3-58191230-G-T	3/281	183/34,013	1.96E-01	1.99	2/55	64/20,734	1.32E-02	12.19	5/336	247/54,747	1.97E-02	3.33
FCN2	**9-137779251-G-A**	**9/281**	**277/34,009**	**6.35E-04**	**4.03**	**1/55**	**45/20,707**	**1.15E-01**	**8.50**	**10/336**	**322/54,716**	**4.35E-05**	**5.18**
GFI1	1-92946625-G-C	2/281	307/34,010	1.00E+00	0.79	2/55	44/20,729	6.60E-03	17.74	4/336	351/54,739	1.73E-01	1.87
IFIH1	2-163136505-C-G	12/281	741/33,970	3.64E-02	2.00	1/55	116/20,714	2.67E-01	3.29	13/336	857/54,684	2.99E-03	2.53
IGLL1	22-23915583-T-C	5/281	320/33,999	1.98E-01	1.91	0/55	495/20,689	--	--	5/336	815/54,688	1.00E+00	1.00
IGLL1	22-23915745-G-A	3/281	30/34,014	2.48E-03	12.22	1/55	208/20,723	4.27E-01	1.83	4/336	238/54,737	6.17E-02	2.76
LIG1	19-48643270-C-T	1/281	149/34,020	1.00E+00	0.81	2/55	24/20,730	2.15E-03	32.56	3/336	173/54,750	9.35E-02	2.84
LY9	**1-160769595-AG-A**	**1/281**	**5/33,975**	**4.82E-02**	**24.26**	**2/55**	**0/20,717**	**6.88E-06**	**1,936.21**	**3/336**	**5/54,692**	**1.24E-05**	**98.54**
MCM5	22-35806756-G-A	11/281	381/34,006	4.32E-04	3.60	0/55	36/20,699	--	--	11/336	417/54,705	7.61E-05	4.41
MDC1	6-30673359-T-G[b]	15/281	952/34,002	1.72E-02	1.96	1/55	1,121/20,686	3.70E-01	0.32	16/336	2,073/54,688	3.17E-01	1.27
NQO2	6-3015818-G-A	0/281	2/34,018	--	--	3/55	87/20,724	1.75E-03	13.69	3/336	89/54,742	1.90E-02	5.53
PKHD1	6-51798908-C-T	0/281	1/34,019	--	--	3/55	29/20,731	8.23E-05	41.18	3/336	30/54,750	1.07E-03	16.43
PLCG2	16-81939089-T-C	6/281	346/34,011	7.09E-02	2.12	0/55	37/20,729	--	--	6/336	383/54,740	3.31E-02	2.58
PLCG2	16-81942175-A-G	8/281	515/34,005	8.08E-02	1.91	5/55	357/20,708	2.67E-03	5.70	13/336	872/54,713	3.43E-03	2.49
STXBP2	**19-7712287-G-C**	**4/280**	**168/34,013**	**5.30E-02**	**2.92**	**0/55**	**15/20,728**	**--**	**--**	**4/335**	**183/54,741**	**2.79E-02**	**3.60**
TCIRG1	11-67818269-G-A	0/281	83/34,011	--	--	7/55	828/20,716	6.26E-03	3.50	7/336	911/54,727	5.16E-01	1.26

[a] Candidate PML risk variants were previously reported (1), see Methods; entries in **bold** are also reported in Tables 2-6 (see main text). p-values were calculated using Fisher's Exact Test, OR = Odds Ratio, CI = Confidence Interval. Ethnic-specific (EUR, AFR, EUR+AFR) gnomAD 3.1 population controls were used (see Methods); NFE in gnomAD were used for EUR.

[b] Since the previous report (1), it was determined that 6-30673359-T-G lies within a region whose mappability results in an artificially low allele number for this variant. Hence, a nearby variant, known to be in linkage disequilibrium with 6-30673359-T-G, was used instead: 6-30671312-A-C (GRCh37), rs28994874.

SUPPLEMENTARY TABLE 5 | Statistical analysis of candidate PML risk variants: drug-exposed PML cases vs. gnomAD 3.1 population controls and drug-exposed controls.[a]

Gene symbol	Variant (GRCh37, hg19)	Drug-exposed PML cases[b]	gnomAD subjects[c]	p-value	OR	OR 95% CI	Drug-exposed controls[d]	p-value	OR	OR 95% CI
AIRE	21-45708278-G-A	0/110	36/76,111	--	--	--	0/718	--	--	--
C8B	**1-57409459-C-A**	**4/110**	**659/76,081**	**1.59E-02**	**4.32**	**1.59 - 11.75**	**4/718**	**1.35E-02**	**6.74**	**1.66 - 27.34**
CFHR2	1-196918605-A-G	0/110	139/76,102	--	--	--	0/718	--	--	--
DNASE1L3	3-58191230-G-T	3/110	1,134/76,109	2.26E-01	1.85	0.59 - 5.85	8/718	1.70E-01	2.49	0.65 - 9.53
FCN2	**9-137779251-G-A**	**5/110**	**511/76,050**	**9.56E-04**	**7.04**	**2.86 - 17.34**	**6/718**	**9.03E-03**	**5.65**	**1.69 - 18.84**
GFI1	1-92946625-G-C	0/110	393/76,104	--	--	--	6/718	--	--	--
IFIH1	2-163136505-C-G	2/110	1,097/76,008	6.74E-01	1.26	0.31 - 5.13	24/705	5.62E-01	0.53	0.12 - 2.26
IGLL1	22-23915583-T-C	0/110	1,095/76,031	--	--	--	1/715	--	--	--
IGLL1	22-23915745-G-A	1/110	296/76,092	3.49E-01	2.35	0.33 - 16.88	3/715	4.36E-01	2.18	0.22 - 21.12
LIG1	19-48643270-C-T	0/110	421/76,114	--	--	--	6/717	--	--	--
LY9	**1-160769595-AG-A**	**1/110**	**11/76,054**	**1.72E-02**	**63.42**	**8.12 - 495.50**	**0/715**	**1.33E-01**	**19.60**	**0.79 - 484.27**
MCM5	22-35806756-G-A	4/110	657/76,043	1.58E-02	4.33	1.59 - 11.78	14/714	2.84E-01	1.89	0.61 - 5.84
MDC1	6-30673359-T-G[e]	6/110	2,864/76,001	3.13E-01	1.47	0.65 - 3.36	26/710	4.22E-01	1.52	0.61 - 3.78
NQO2	6-3015818-G-A	0/110	96/76,105	--	--	--	0/717	--	--	--
PKHD1	6-51798908-C-T	0/110	31/76,115	--	--	--	0/718	--	--	--
PLCG2	16-81939089-T-C	1/110	511/76,104	5.24E-01	1.36	0.19 - 9.74	9/718	1.00E+00	0.72	0.09 - 5.76
PLCG2	16-81942175-A-G	3/110	1,187/76,054	2.47E-01	1.77	0.56 - 5.58	25/718	1.00E+00	0.78	0.23 - 2.62
STXBP2	**19-7712287-G-C**	**2/110**	**208/76,099**	**3.73E-02**	**6.76**	**1.66 - 27.55**	**0/718**	**1.75E-02**	**33.11**	**1.58 - 694.35**
TCIRG1	11-67818269-G-A	0/110	1,072/76,089	--	--	--	10/718	--	--	--

[a] Candidate PML risk variants were previously reported (1), see Methods; entries in **bold** are also reported in Tables 2-6 (see main text). p-values were calculated using Fisher's Exact Test, OR = Odds Ratio, CI = Confidence Interval.

[b] Variant-positive drug-exposed PML cases includes the following MS drugs: 1 dimethyl fumarate, 20 natalizumab, 3 rituximab, and 2 MS patients with an unknown drug (see Methods).

[c] All ethnicities in gnomAD 3.1 population controls were used (see Methods).

[d] Variant-positive drug-exposed JCV+ MS controls (i.e., matched controls, see Methods) includes the following MS drugs: 6 dimethyl fumarate, 6 fingolimod, 105 natalizumab, 1 ocrelizumab, and 4 rituximab.

[e] Since the previous report (1), it was determined that 6-30673359-T-G lies within a region whose mappability results in an artificially low allele number for this variant. Hence, a nearby variant, known to be in linkage disequilibrium with 6-30673359-T-G, was used instead: 6-30671312-A-C (GRCh37), rs28994874.

SUPPLEMENTARY TABLE 6 | Statistical analysis of candidate PML risk variants: NTZ-exposed PML cases vs. gnomAD 3.1 population controls and NTZ-exposed controls.[a]

Gene	Variant (GRCh37)	NTZ-exposed PML cases[b]	gnomAD subjects[c]	p-value	OR	OR 95% CI	NTZ-exposed JCV+ MS controls[d]	p-value	OR	OR 95% CI
AIRE	21-45708278-G-A	0/86	36/76,111	--	--	--	0/604	--	--	--
C8B	**1-57409459-C-A**	**4/86**	**659/76,081**	**6.88E-03**	**5.58**	**2.04 - 15.28**	**3/604**	**5.88E-03**	**9.77**	**2.15 - 44.44**
CFHR2	1-196918605-A-G	0/86	139/76,102	--	--	--	0/604	--	--	--
DNASE1L3	3-58191230-G-T	2/86	1,134/76,109	3.68E-01	1.57	0.39 - 6.41	6/604	2.62E-01	2.37	0.47 - 11.95
FCN2	**9-137779251-G-A**	**2/86**	**511/76,050**	**1.15E-01**	**3.52**	**0.86 - 14.34**	**4/604**	**1.65E-01**	**3.57**	**0.64 - 19.8**
GFI1	1-92946625-G-C	0/86	393/76,104	--	--	--	6/604	--	--	--
IFIH1	2-163136505-C-G	2/86	1,097/76,008	3.53E-01	1.63	0.4 - 6.62	22/593	7.56E-01	0.62	0.14 - 2.68
IGLL1	22-23915583-T-C	0/86	1,095/76,031	--	--	--	1/602	--	--	--
IGLL1	22-23915745-G-A	0/86	296/76,092	--	--	--	1/601	--	--	--
LIG1	19-48643270-C-T	0/86	421/76,114	--	--	--	3/603	--	--	--
LY9	**1-160769595-AG-A**	**1/86**	**11/76,054**	**1.35E-02**	**81.33**	**10.38 - 636.94**	**0/602**	**1.25E-01**	**21.14**	**0.85 - 523.14**
MCM5	22-35806756-G-A	3/86	657/76,043	3.91E-02	4.15	1.31 - 13.16	13/600	4.39E-01	1.63	0.46 - 5.85
MDC1	6-30673359-T-G[e]	6/86	2,864/76,001	1.43E-01	1.92	0.83 - 4.39	24/596	2.53E-01	1.79	0.71 - 4.51
NQO2	6-3015818-G-A	0/86	96/76,105	--	--	--	0/603	--	--	--
PKHD1	6-51798908-C-T	0/86	31/76,115	--	--	--	0/604	--	--	--
PLCG2	16-81939089-T-C	1/86	511/76,104	4.40E-01	1.74	0.24 - 12.52	9/604	1.00E+00	0.78	0.10 - 6.22
PLCG2	16-81942175-A-G	3/86	1,187/76,054	1.52E-01	2.28	0.72 - 7.22	24/604	1.00E+00	0.87	0.26 - 2.96
STXBP2	**19-7712287-G-C**	**2/86**	**208/76,099**	**2.38E-02**	**8.69**	**2.12 - 35.54**	**0/604**	**1.54E-02**	**35.77**	**1.70 - 751.47**
TCIRG1	11-67818269-G-A	0/86	1,072/76,089	--	--	--	10/604	--	--	--

[a] Candidate PML risk variants were previously reported (1), see Methods; association results are reported for the natalizumab (NTZ) subset; variants in **bold** are reported in Tables 2-6 for all drug-exposed (see main text). p-values were calculated using Fisher's Exact Test, OR = Odds Ratio, CI = Confidence Interval.

[b] Variant-positive NTZ-exposed PML cases includes 20 NTZ.

[c] All ethnicities in gnomAD 3.1 population controls were used (see Methods).

[d] Variant-positive NTZ-exposed JCV+ MS controls (see Methods) includes 105 NTZ and a subset of these were also exposed to other MS drugs: 4 dimethyl fumarate, 6 fingolimod, and 4 rituximab.

[e] Since the previous report (1), it was determined that 6-30673359-T-G lies within a region whose mappability results in an artificially low allele number for this variant. Hence, a nearby variant, known to be in linkage disequilibrium with 6-30673359-T-G, was used instead: 6-30671312-A-C (GRCh37), rs28994874.

SUPPLEMENTARY TABLE 7 | Candidate PML risk variant assessment for association with MS.[a]

Gene symbol	Variant (GRCh37, hg19)[b]	dbSNP ID	HumanExome Beadchip Variant ID[c]	p-value	OR
AIRE	21-45708278-G-A	rs148012328	not on chip	not on chip	not on chip
C8B	**1-57409459-C-A**	**rs139498867**	**exm63348**	**0.06695**	**0.9669**
CFHR2	1-196918605-A-G	rs148175483	exm133701	data not reported	data not reported
DNASE1L3	3-58191230-G-T	rs12491947	exm326513	0.68340	0.9896
FCN2	**9-137779251-G-A**	**rs76267164**	**exm795364**	**0.70880**	**1.0085**
GFI1	1-92946625-G-C	rs149914857	exm75192	0.30500	0.9561
IFIH1	2-163136505-C-G	rs35337543	exm236925	0.54950	0.9917
IGLL1	22-23915583-T-C	rs1064421	not on chip	not on chip	not on chip
IGLL1	22-23915745-G-A	rs143780139	not on chip	not on chip	not on chip
LIG1	19-48643270-C-T	rs3730947	exm1486285	0.70230	0.9793
LY9	**1-160769595-AG-A**	**rs763811636**	**not on chip**	**not on chip**	**not on chip**
MCM5	22-35806756-G-A	rs2230933	exm1603516	0.08046	0.9650
MDC1	6-30673359-T-G[d]	rs143258964	not on chip	data not reported	data not reported
NQO2	6-3015818-G-A	rs148024596	exm513064	data not reported	data not reported
PKHD1	6-51798908-C-T	rs199589074	exm554871	0.93770	0.9741
PLCG2	16-81939089-T-C	rs187956469	exm1262450	0.02654	0.9420
PLCG2	16-81942175-A-G	rs75472618	exm1262467	0.35370	0.9837
STXBP2	**19-7712287-G-C**	**rs35490401**	**exm1416872**	**0.68530**	**0.9889**
TCIRG1	11-67818269-G-A	rs75596506	exm934645	0.61910	0.9781

[a] MS association data were previously reported (8), see Methods.

[b] Candidate PML risk variants were previously reported (1), see Methods; entries in **bold** are also reported in Tables 2-6 (see main text).

[c] A subset of variants (4 of 19) were not present on the Beadchip; two variants (exm133701 and exm513064) were present on the Beadchip but p-values and ORs were not reported.

[d] Since the previous report (1), it was determined that 6-30673359-T-G lies within a region whose mappability results in an artificially low allele number for this variant. Hence, a nearby variant, known to be in linkage disequilibrium with 6-30673359-T-G, was used instead: 6-30671312-A-C (GRCh37), rs28994874.

SUPPLEMENTARY TABLE 8 | Candidate PML risk variant distribution across ethnicities, PML cohorts, primary diseases, and drug exposures.[a]

Gene symbol	Variant (GRCh37, hg19)	dbSNP ID	Ethnicity[b]		Primary disease[c]	Drug exposure[d]
			Primary	Secondary		
AIRE	21-45708278-G-A	rs148012328	2 AFR	1 EUR	2 HIV	2 n/a
C8B	**1-57409459-C-A**	**rs139498867**	**7 EUR, 2 AFR**	**1 ASJ/Other, 1 EUR/Other, 1 AMR**	**1 BC, 4 HIV, 4 MS**	**4 NTZ, 1 unk, 4 n/a**
CFHR2	1-196918605-A-G	rs148175483	3 AFR	1 AMR/EUR	3 HIV	3 n/a
DNASE1L3	3-58191230-G-T	rs12491947	3 EUR, 2 AFR	1 AMR/EUR, 1 AMR/EUR/SAS	2 HIV, 3 MS	2 NTZ, 1 unk, 2 n/a
FCN2	**9-137779251-G-A**	**rs76267164**	**9 EUR, 1 AFR**	**1 AFR, 1 EUR, 1 AFR/ASJ/Other**	**2 BC, 4 HIV, 3 MS, 1 Other**	**1 DMF, 2 NTZ, 2 RTX, 1 unk, 4 n/a**
GFI1	1-92946625-G-C	rs149914857	2 EUR, 2 AFR	1 EUR/Other	1 BC, 3 HIV	1 unk, 3 n/a
IFIH1	2-163136505-C-G	rs35337543	12 EUR, 1 AFR	1 AFR, 1 EUR, 1 FIN, 2 Other	1 BC, 9 HIV, 2 MS, 1 Other	2 NTZ, 2 unk, 9 n/a
IGLL1	22-23915583-T-C	rs1064421	5 EUR	none	4 BC, 1 HIV	4 unk, 1 n/a
IGLL1	22-23915745-G-A	rs143780139	3 EUR, 1 AFR	none	3 HIV, 1 MS	1 unk, 3 n/a
LIG1	19-48643270-C-T	rs3730947	1 EUR, 2 AFR	1 AFR, 1 EUR, 1 SAS/Other	3 HIV	3 n/a
LY9	**1-160769595-AG-A**	**rs763811636**	**1 EUR, 2 AFR**	**2 EUR/Other, 1 SAS/Other**	**1 HIV, 1 MS, 1 Other**	**1 NTZ, 1 unk, 1 n/a**
MCM5	22-35806756-G-A	rs2230933	11 EUR	1 AFR/AMR, 1 AFR/Other, 1 Other	5 HIV, 3 MS, 3 Other	3 NTZ, 1 RTX, 2 unk, 5 n/a
MDC1	6-30673359-T-G[e]	rs143258964	15 EUR, 1 AFR	1 AFR, 1 AFR/AMR, 1 SAS/Other	1 BC, 8 HIV, 6 MS, 1 Other	6 NTZ, 2 unk, 8 n/a
NQO2	6-3015818-G-A	rs148024596	3 AFR	1 EUR	3 HIV	3 n/a
PKHD1	6-51798908-C-T	rs199589074	3 AFR	none	3 HIV	3 n/a
PLCG2[f]	16-81939089-T-C	rs187956469	6 EUR	1 AFR	4 HIV, 1 MS, 1 Other	1 NTZ, 1 unk, 4 n/a
PLCG2[f]	16-81942175-A-G	rs75472618	8 EUR, 5 AFR	1 AFR, 2 EUR, 2 EUR/Other	9 HIV, 3 MS, 1 Other	3 NTZ, 1 unk, 9 n/a
STXBP2	**19-7712287-G-C**	**rs35490401**	**4 EUR**	**1 AFR, 1 Other**	**1 HIV, 2 MS, 1 Other**	**2 NTZ, 1 unk, 1 n/a**
TCIRG1	11-67818269-G-A	rs75596506	7 AFR	4 EUR/Other	7 HIV	7 n/a

[a] Candidate PML risk variants were previously reported (1), see Methods; entries in **bold** are also reported in Tables 2-6 (see main text).

[b] All PML cases were assigned a Primary Ethnicity of EUR or AFR and a Secondary Ethnicity is reported for the subset of PML cases in which another ethnicity was present at >= 5%; see Methods for details.

[c] Primary disease: BC = blood cancer, HIV = human immunodeficiency virus infected, MS = multiple sclerosis, Other = various; see Methods for list of diseases under the BC and Other subgroups.

[d] Drug Exposure: DMF = dimethyl fumarate, NTZ = natalizumab, RTX = rituximab, unk = unknown, n/a = not applicable (i.e., PML cases with HIV as their primary disease).

[e] Since the previous report (1), it was determined that 6-30673359-T-G lies within a region whose mappability results in an artificially low allele number for this variant. Hence, a nearby variant, known to be in linkage disequilibrium with 6-30673359-T-G, was used instead: 6-30671312-A-C (GRCh37), rs28994874.

[f] For the pair of PLCG2 variants, 6/13 PML cases have both 16-81939089-T-C and 16-81942175-A-G.

SUPPLEMENTARY TABLE 9 | Association statistics[a] and variant frequency for top 4 PML risk variants: PML cases vs. controls.[b]

	Variant genome position (GRCh37, hg19), Gene symbol				
	**1-57409459-C-A** ***C8B***	**9-137779251-G-A** ***FCN2***	**1-160769595-AG-A** ***LY9***	**19-7712287-G-C** ***STXBP2***	**4-variant panel**
**All**					
PML cases	9/336	10/336	3/336	4/335	26/336
Frequency in cases	2.7%	3.0%	0.9%	1.2%	7.7%
gnomAD 3.1 controls	659/76,081	511/76,050	11/76,054	208/76,099	1,389/76,071
Frequency in controls	0.9%	0.7%	0.01%	0.3%	1.8%
p-value	3.08E-03	1.23E-04	2.96E-05	1.46E-02	1.36E-09
OR	3.15	4.53	62.28	4.41	4.51
OR 95% CI	1.62 - 6.14	2.40 - 8.56	17.30 - 224.25	1.63 - 11.93	3.01 - 6.75
**Drug-exposed**					
PML cases	4/110	5/110	1/110	2/110	12/110
Frequency in cases	3.6%	4.5%	0.9%	1.8%	10.9%
gnomAD 3.1 controls	659/76,081	511/76,050	11/76,054	208/76,099	1,389/76,071
Frequency in controls	0.9%	0.7%	0.01%	0.3%	1.8%
p-value	1.59E-02	9.56E-04	1.72E-02	3.73E-02	9.60E-07
OR	4.32	7.04	63.42	6.76	6.58
OR 95% CI	1.59 - 11.75	2.86 - 17.34	8.12 - 495.5	1.66 - 27.55	3.61 - 12.02
Matched controls	4/718	6/718	0/715	0/718	10/718
Frequency in controls	0.6%	0.8%	0%	0%	1.4%
p-value	1.35E-02	9.03E-03	1.33E-01	1.75E-02	3.50E-06
OR	6.74	5.65	19.60	33.11	8.67
OR 95% CI	1.66 - 27.34	1.69 - 18.84	0.79 - 484.27	1.58 - 694.35	3.65 - 20.60
**Natalizumab-exposed**					
PML cases	4/86	2/86	1/86	2/86	9/86
Frequency in cases	4.7%	2.3%	1.2%	2.3%	10.5%
gnomAD 3.1 controls	659/76,081	511/76,050	11/76,054	208/76,099	1,389/76,071
Frequency in controls	0.9%	0.7%	0.01%	0.3%	1.8%
p-value	6.88E-03	1.15E-01	1.35E-02	2.38E-02	2.99E-05
OR	5.58	3.52	81.33	8.69	6.28
OR 95% CI	2.04 - 15.28	0.86 - 14.34	10.38 - 636.9	2.12 - 35.54	3.14 - 12.56
Matched controls	3/604	4/604	0/602	0/604	7/604
Frequency in controls	0.5%	0.7%	0%	0%	1.2%
p-value	5.88E-03	1.65E-01	1.25E-01	1.54E-02	2.68E-05
OR	9.77	3.57	21.14	35.77	9.97
OR 95% CI	2.15 - 44.44	0.64 - 19.80	0.85 - 523.1	1.70 - 751.5	3.61 - 27.53

[a] p-values were calculated using Fisher's Exact Test, OR = Odds Ratio, CI = Confidence Interval.

[b] For controls, gnomAD 3.1 subjects were used as population controls and drug-exposed JCV+ MS patients were used as matched controls.
